# Efficacy of WeChat-Based Digital Intervention Versus Metformin in Women With Polycystic Ovary Syndrome: Randomized Controlled Trial

**DOI:** 10.2196/55883

**Published:** 2024-10-02

**Authors:** Diliqingna Dilimulati, Xiaowen Shao, Lihua Wang, Meili Cai, Yuqin Zhang, Jiayi Lu, Yao Wang, Hongying Liu, Ming Kuang, Haibing Chen, Manna Zhang, Shen Qu

**Affiliations:** 1 Department of Endocrinology and Metabolism Shanghai Tenth People’s Hospital Tongji University School of Medicine Shanghai China; 2 Department of Obstetrics and Gynecology Shanghai Tenth People’s Hospital Tongji University School of Medicine Shanghai China; 3 Department of Endocrinology and Metabolism Renji Hospital Shanghai Jiaotong University School of Medicine Shanghai China; 4 Department of Clinical Laboratory Shanghai Tenth People’s Hospital Tongji University School of Medicine Shanghai China; 5 Department of Dermatology Shanghai Tenth People’s Hospital Tongji University School of Medicine Shanghai China; 6 Hangzhou Kang Ming Information Technology Co., Ltd Hangzhou China

**Keywords:** polycystic ovary syndrome, insulin resistance, digital intervention, metformin, women’s health

## Abstract

**Background:**

The first-line treatment for polycystic ovary syndrome (PCOS) is lifestyle modification. However, it is currently unknown whether digital medicine can assist patients with PCOS in maintaining a healthy lifestyle while alleviating PCOS symptoms.

**Objective:**

This study aims to evaluate the efficacy of WeChat-based digital intervention versus metformin treatment in women with PCOS and insulin resistance.

**Methods:**

A total of 80 women with PCOS and insulin resistance were recruited from an endocrinology clinic and randomly assigned to receive either a WeChat-based digital intervention (n=40, 50%) or metformin (n=40, 50%) for 12 weeks. The WeChat-based digital intervention consisted of 3 modules; a coach assisted the patients in using the intervention. The primary outcome was the change in a homeostatic model assessment for insulin resistance. At baseline and after the 12-week intervention, anthropometric parameters, menstruation frequency, sex hormone levels, metabolic factors, and body fat distribution were measured in the clinic. Furthermore, self-assessed web-based questionnaires on diet, exercise, sleep, anxiety, and depression were obtained.

**Results:**

A total of 72 participants completed the follow-up (for a 90% follow-up rate), including 35 of 40 (88%) participants from the digital intervention group and 37 of 40 (93%) participants from the metformin group. The homeostatic model assessment for insulin resistance in the digital intervention group was significantly improved after 12 weeks of treatment with a mean change of –0.93 (95% CI –1.64 to –0.23), but no statistical difference was observed between the groups (least squares mean difference –0.20; 95% CI –0.98 to 0.58; *P*=.62). Both digital intervention and metformin treatment significantly improved menstruation frequency (digital intervention: *P*<.001; metformin: *P*<.001) and reduced body weight (digital intervention: *P*<.001; metformin: *P*<.001) and total fat mass (digital intervention: *P*<.001; metformin: *P*<.001). Furthermore, the digital intervention had a significant advantage over metformin in improving waist circumference (least squares mean difference –1.84; 95% CI –3.44 to –0.24; *P*=.03), waist-to-hip ratio (least squares mean difference –0.02; 95% CI –0.03 to 0.00; *P*=.03), total fat mass (least squares mean difference –1.59; 95% CI –2.88 to –0.30; *P*=.02), and dehydroepiandrosterone sulfate (least squares mean difference –69.73; 95% CI –129.70 to –9.75; *P*=.02). In terms of safety, the main adverse events were sensations of hunger in the digital intervention group (2/40, 5%) and gastrointestinal adverse events in the metformin group (12/40, 30%).

**Conclusions:**

Our data suggest that digital intervention is an effective treatment option for patients with PCOS, with an efficacy comparable to that of metformin, and that it can also alleviate the negative effects of medications and make it easier and more efficient to adhere to lifestyle treatments. WeChat-based digital interventions have the potential to provide a new path for the improvement and health of women with PCOS in China.

**Trial Registration:**

ClinicalTrials.gov NCT05386706; https://clinicaltrials.gov/study/NCT05386706

## Introduction

### Background

Approximately 10% to 13% of women of reproductive age worldwide have polycystic ovary syndrome (PCOS) [1]. The prevalence of PCOS is increasing globally because of changes in environmental variables and lifestyle, as well as increased disease awareness and detection rates. PCOS is characterized by a range of heterogeneous symptoms with varying severities, including abnormal menstruation, hyperandrogenism (hirsutism or hyperandrogenemia), and ovarian dysfunction (oligo-ovulation or polycystic ovary morphology) [2]. One of the most prevalent endocrine disorders among patients with PCOS is insulin resistance, with variable degrees of insulin resistance present in approximately 75% of patients with PCOS [3]. For such patients, insulin resistance reduction is essential in the management of various endocrine and metabolic disorders [4].

The first-line treatment for PCOS is lifestyle intervention, and studies have demonstrated that positive lifestyle changes can improve hyperandrogenemia, menstrual cycle, hirsutism, and insulin resistance in the affected patients [5]. However, lifestyle intervention is associated with poor compliance and low sustainability. Nevertheless, there are no indicators to determine whether patients are making enough progress in improving their lifestyle. Studies have demonstrated that digital therapy accessed on mobile phones, which are convenient and accessible, can effectively improve behavioral changes in diet, exercise, and medication adherence while predicting disease progression, reducing the frequency of disease-related symptoms, and promoting effective disease management [6,7]. Regarding PCOS, which is a chronic disease, there are limited digital treatment methods. A previous 3-component lifestyle intervention consisting of diet, exercise, and cognitive behavioral therapy, facilitated using texting to track patients’ food intake, physical activity, and mood, can effectively improve eating disorder and mood in patients with PCOS and contribute to weight loss [8,9]. This study focused on the effect of digital intervention compared with metformin in improving homeostatic model assessment for insulin resistance (HOMA-IR) levels and other metabolic and reproductive indicators.

### Objectives

This was a single-center, prospective, randomized controlled clinical trial designed to determine whether digital intervention was effective in patients with PCOS after 12 weeks of treatment. Evaluations were conducted on improvements in PCOS-related clinical parameters and side effects for both groups. Simultaneously, a patient satisfaction survey was conducted for the digital intervention. This study aimed to confirm the efficacy and safety of digital intervention in the treatment of PCOS.

## Methods

### Study Design and Participants

A total of 80 women with PCOS (including 73 newly diagnosed and 7 previously diagnosed women with PCOS) from the endocrinology clinic of a tertiary affiliated hospital of Tongji University were enrolled between June 21, 2022, and August 12, 2023, in this single-center, unblinded, 2-arm parallel study with 1:1 allocation. PCOS was diagnosed in patients attending PCOS outpatient clinics, and eligible patients were invited to participate in this study. The CONSORT eHEALTH (Consolidated Standards of Reporting Trials of Electronic and Mobile Health Applications and Online Telehealth) checklist is presented in Multimedia Appendix 1.

The inclusion criteria were being aged 18 to 45 years, having a clinical diagnosis of PCOS according to the 2003 Rotterdam diagnostic criteria [10], and having a HOMA-IR score ≥1.8 (in Asian countries, a HOMA-IR score ≥1.8 can diagnose insulin resistance and predict the occurrence of metabolic syndrome [11]). The exclusion criteria included the inability to use WeChat; hyperthyroidism or hypothyroidism; severe abnormal liver function (liver enzyme levels >3 times the standard limit); severe abnormal renal function (serum creatinine level >123.8 μmol/L or estimated glomerular filtration rate <45 mL/min/1.73 m^2^); congenital adrenal hyperplasia, hyperprolactinemia, or adrenal tumor; severe infection, severe anemia, neutropenia, and other blood system diseases; type 1 diabetes, monogenic mutation diabetes, or diabetes due to pancreatic damage or other secondary diabetes; existence of mental illness or dementia; history of using contraceptives, metformin, glucagon-like peptide-1 analogs, pioglitazone, all classes of antidepressant medications, and other drugs in the past 3 months; pregnancy or intention to become pregnant within 6 months; participation in another clinical trial within 3 months; and declining to participate. Moreover, perimenopausal or menopausal women were excluded from this study. As this last exclusion criterion was not reported in the ClinicalTrials.gov registration (NCT05386706), this exclusion was a protocol deviation.

### Ethical Considerations

The study protocol was approved by the ethics committee of the Shanghai Tenth People’s Hospital (SHSY-IEC-5.0/21K191/P02); the study was registered in ClinicalTrials.gov (NCT05386706); and the name of the trial registry is Digital Intervention PCOS. No changes were made to this clinical trial after registration. All participants provided a signed written informed consent. Every participant was informed that their participation in the study was completely voluntary, that the information gathered for the study would be kept private, and that they could revoke their agreement at any moment without it having an adverse effect on their care. Each participant was given a unique study ID for data entry, maintenance, and analysis, and their personal information was anonymized.

### Randomization and Procedure

Following baseline evaluation, based on a predefined, computer-generated number in a 1:1 assignment, the participants were randomly assigned to receive either digital intervention or metformin. Allocation concealment was achieved by using opaque, sealed envelopes with sequential numbers. One researcher created the random number sequence for this investigation, and another researcher enrolled and assigned participants. During intervention, both groups were aware of their allocation. After intervention, the percentage of adherence to the intervention was calculated as adherence days divided by intervention days.

### Digital Intervention Group

**Figure 1:**
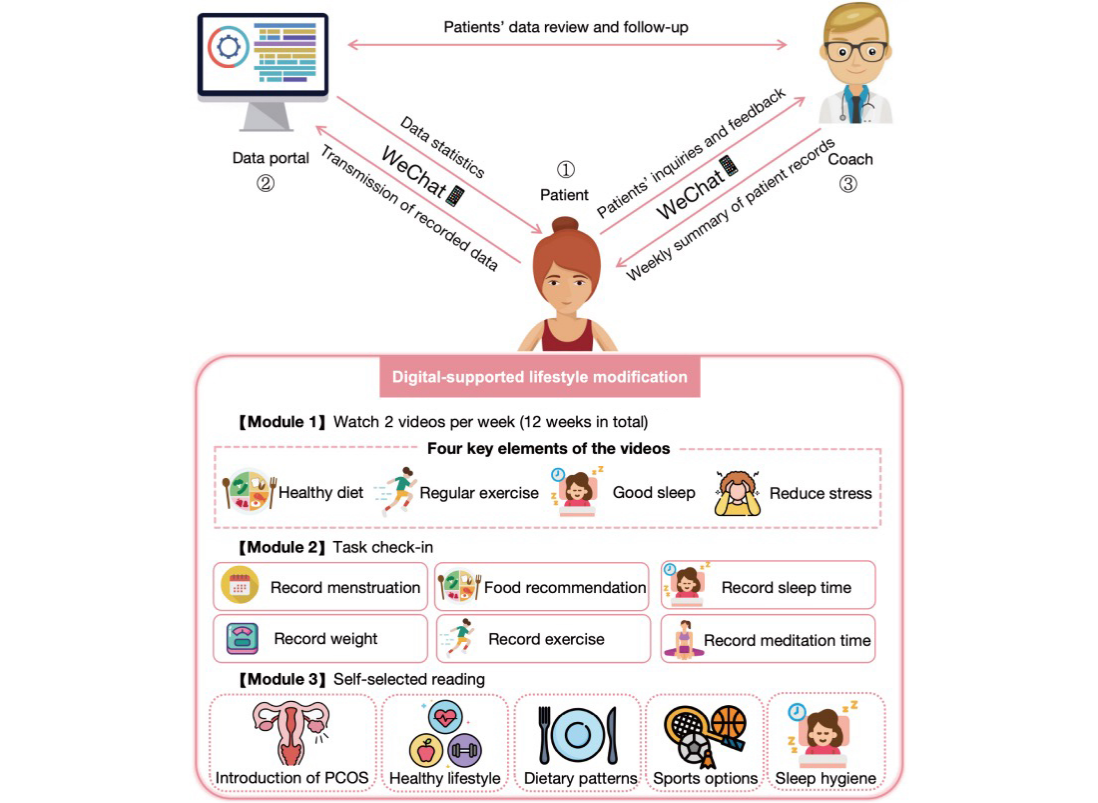
Overview of the components of the digital intervention.

The PCOS guidelines recommend lifestyle interventions as the first-line treatment for PCOS (diet, exercise, sleep, and mood) [12,13]. Cognitive behavioral therapy is a type of treatment that combines behavioral and cognitive methods [14]. Prior studies evaluated the effectiveness of 20 cognitive behavioral therapy lifestyle courses with physical therapy, healthy eating, and 9 months of extra feedback via a mobile SMS text messaging service to conventional treatment [8]. In this regard, we designed a digital intervention method based on the WeChat mini program. The mini program was pretested with 10 users for usability and face validity before being used in the intervention. The WeChat mini program was designed by the Endocrinology and Metabolism Department of Shanghai Tenth People’s Hospital and developed by Shanghai Zoey Information Technology Co, Ltd. Patients with PCOS assigned to the digital intervention group can register a new account for free through WeChat, and they need to use the WeChat mini program for 12 weeks. As presented in Figure 1, the program consists of 3 modules. In module 1, patients can watch 2 videos per week, with each video being about 5 minutes long; a total of 24 videos can be watched over a 12-week period. These videos describe the 4 elements of a healthy lifestyle that should be prioritized by patients with PCOS: healthy diet, regular exercise, good sleep, and stress reduction. The video specifically explains how the 4 elements affect the occurrence and development of PCOS, what can be effectively done to achieve a good lifestyle, and how to adhere to these lifestyles. Multimedia Appendix 2 presents the title and main content of each video. In module 2, patients need to record their menstruation during their menstrual period (duration of menstruation, amount of bleeding, body temperature, and mood) and record their daily weight (weight change value, BMI, and weight status appearing after weight submission); daily exercise time (according to different exercise intensities, low-, moderate-, and high-intensity exercises were recorded, and the type of exercise needed to be selected); daily sleep time (bedtime and wake-up time); and daily meditation time (also provides 24 pieces of meditation music). Simultaneously, the list of recommended food and food to avoid is provided in the food recommendation part. After recording, the patients can see a statistical chart of their weight and exercise in module 2. To allow patients to sufficiently understand the important role of lifestyle improvement in PCOS treatment, we have set up module 3, which they can selectively read by themselves, including an overall introduction to PCOS; healthy lifestyles suitable for PCOS; different dietary patterns (including low-carbohydrate, high-protein, and restricted energy-balanced diets); choices of different exercises (the intensity of each exercise and the amount of energy that can be consumed); and importance of sleep hygiene (the dangers of circadian disruption). Meanwhile, contact between the patient and doctor through WeChat’s electronic message was synchronized, and communication was scheduled every Monday. The doctor acts as the patient’s coach, downloading the patient’s record data every week; analyzing the patient’s performance that week; summarizing the weekly exercise time, weight, sleep, and menstrual period and sending it to the patient; and suggesting possible improvements. In addition, the doctor asks the patients if they encountered problems that cannot be solved and provides support and encouragement. While using the digital intervention, 5 patients experienced temporary system problems (4 patients had video playback issues and 1 patient had data entry issues). After communicating with the programmer, we optimized and improved the system in time and solved the problems encountered by the patients, but the content has not changed.

### Metformin Group

Metformin, which improves ovulation and enhances insulin sensitivity, is the first-line insulin-sensitizing medication used to treat PCOS; it was used as a treatment drug in the control group in this study [15]. Metformin should be used for at least 3 months after completion depending on the patient’s ovulation recovery, androgen status, and metabolic clinical indicators [16,17]. During the 12-week intervention, the metformin group was administered 1000 mg of metformin (Glucophage, Merck KGaA) per day. In previous clinical trials, the dose of metformin ranged from 850 mg to 2000 mg per day [18]. To reduce the side effects of drugs, the dose of 1000 mg per day was selected for this study, and a doctor educated the patients regarding healthy lifestyles during the first visit.

### Measurements

#### Clinical and Laboratory Measurements

The primary outcome was HOMA-IR alterations, whereas the secondary outcomes were changes in menstrual cycles, metabolic indicators, sex hormones, body composition, and degree of fibrosis and steatosis in the liver. Furthermore, body weight, waist circumference, and hip circumference were collected. Body weight (kg)/height (m^2^) was used to determine the BMI, and waist circumference (cm)/hip circumference (cm) was used to determine the waist-to-hip ratio. Menstrual cycle is defined as the total number of menstruations in the past 12 months. The following metabolic variables were assessed: fasting blood glucose, fasting insulin, total cholesterol, triglycerides, high-density lipoprotein cholesterol, low-density lipoprotein cholesterol, alanine aminotransferase (ALT), aspartate aminotransferase (AST), creatinine, uric acid, sex hormone–binding globulin (SHBG), and glycated hemoglobin A1c (HbA_1c_). A 75-g oral glucose tolerance test was used to evaluate the postprandial blood glucose and postprandial insulin levels at 120 minutes. Sex hormones included the luteinizing hormone (LH), follicle-stimulating hormone, total testosterone, free testosterone, androstenedione, anti-Müllerian hormone, and dehydroepiandrosterone sulfate (DHEAS). (Fasting blood glucose [mmol/L]×fasting insulin [mU/L])/22.5 was used to calculate the HOMA-IR levels [19]. Transient elastography (Echosens) was used to measure the controlled attenuation parameter and determine the liver stiffness measurement, which represent the extent of liver steatosis and fibrosis, respectively. Dual-energy x-ray absorptiometry (Hologic) was used to measure body composition. At baseline and after the 12-week intervention, all clinical and laboratory tests were conducted at the clinic.

#### Questionnaires

At baseline and after the 12-week intervention, all participants were required to complete the self-assessed web-based questionnaires, including the 21-item Three-Factor Eating Questionnaire (TFEQ-R21), International Physical Activity Questionnaire (IPAQ), Pittsburgh Sleep Quality Index (PSQI), and Hospital Anxiety and Depression Scale (HADS). Furthermore, participants in the digital intervention group filled out a self-assessed web-based satisfaction survey 12 weeks after the intervention, and the 5 questions in the questionnaire are shown in Multimedia Appendix 3.

TFEQ-R21 is a questionnaire used to examine eating behavior [20]. The 3 scales comprising the TFEQ-R21 are the cognitive restraint scale (6 items), uncontrolled eating scale (9 items), and emotional eating scale (6 items). These scales cover different aspects of eating behavior. The higher the score, the more severe the behavior of cognitive restraint, uncontrolled eating, or emotional eating.

The translated short form of the IPAQ was used to self-evaluate physical activity, and there was a 7-day recall period. The usefulness of the IPAQ has been validated. It provides data on the amount of time spent each week on several physical activity domains, including low, moderate, and vigorous intensity [21,22]. Metabolic equivalent (MET) is a measure of energy expenditure expressed as a multiple of resting energy costs and can be used to quantify the intensity of different activities. By calculating MET×days×daily time, standardized techniques were used to convert time, days per activity, and intensity to MET minute per week scores. One minute of walking, 1 minute of general moderate-intensity activity, and 1 minute of vigorous exercise were equivalent to 3.3, 4.0, and 8.0 METs, respectively.

The PSQI is a self-report assessment tool that measures sleep quality over a 30-day period [23]. The scale yields a global score and 7 component scores, namely, subjective sleep quality, sleep latency, duration, efficiency, sleep disruptions, use of sleeping pills, and dysfunction throughout the day. Every element receives a score between 0 and 3, and the overall result can range from 0 to 21. A higher number indicates lower sleep quality.

A 14-item test called the HADS is used to assess an individual’s anxiety and depression levels [24]. Of 14 items, 7 are associated with depression, and the remaining items are associated with anxiety. The 4 response possibilities on each item range from 0 to 3, enabling the subscales of anxiety and depression (HADS-A and HADS-D) to have independent total scores, each ranging from 0 to 21. While the scores of 0 to 7 are considered as “normal” and 8 to 10 are considered as “borderline,” scores of 11 or more on each subscale are considered to indicate serious instances of psychological morbidity.

#### Sample Size Estimation

On the basis of previous research [17,25,26], we calculated the mean difference in the HOMA-IR levels before and after the metformin intervention as 1.0 and the SD of the outcome variable as 1.0. Alternatively, we predicted the mean difference in the HOMA-IR levels before and after the digital intervention to be 2.0 and the SD of the outcome variable to be 1.0. Our power analysis revealed that according to a web-based calculator (MedSci Sample Size tools), with a 30% sample loss rate, a minimum of 30 participants were required for each group to achieve a significant outcome (α=.05, power=0.90); the total sample size of the study was 60.

#### Statistical Analysis

The intention-to-treat principle was used for all data analyses, and SPSS (version 25; IBM Corp) was used for all statistical analyses. Continuous variables were expressed as means with 95% CIs. Mean differences between the two treatment groups are displayed as least squares mean changes and 95% CIs. To evaluate the group differences in the primary and continuous secondary end points, we used an analysis of covariance model in which the treatment was a fixed effect and the corresponding baseline value was a covariate. Fisher exact test or chi-square test was used where applicable to analyze categorical variables. A *P* value <.05 was considered to be statistically significant.

## Results

### Participant Characteristics

**Figure 2:**
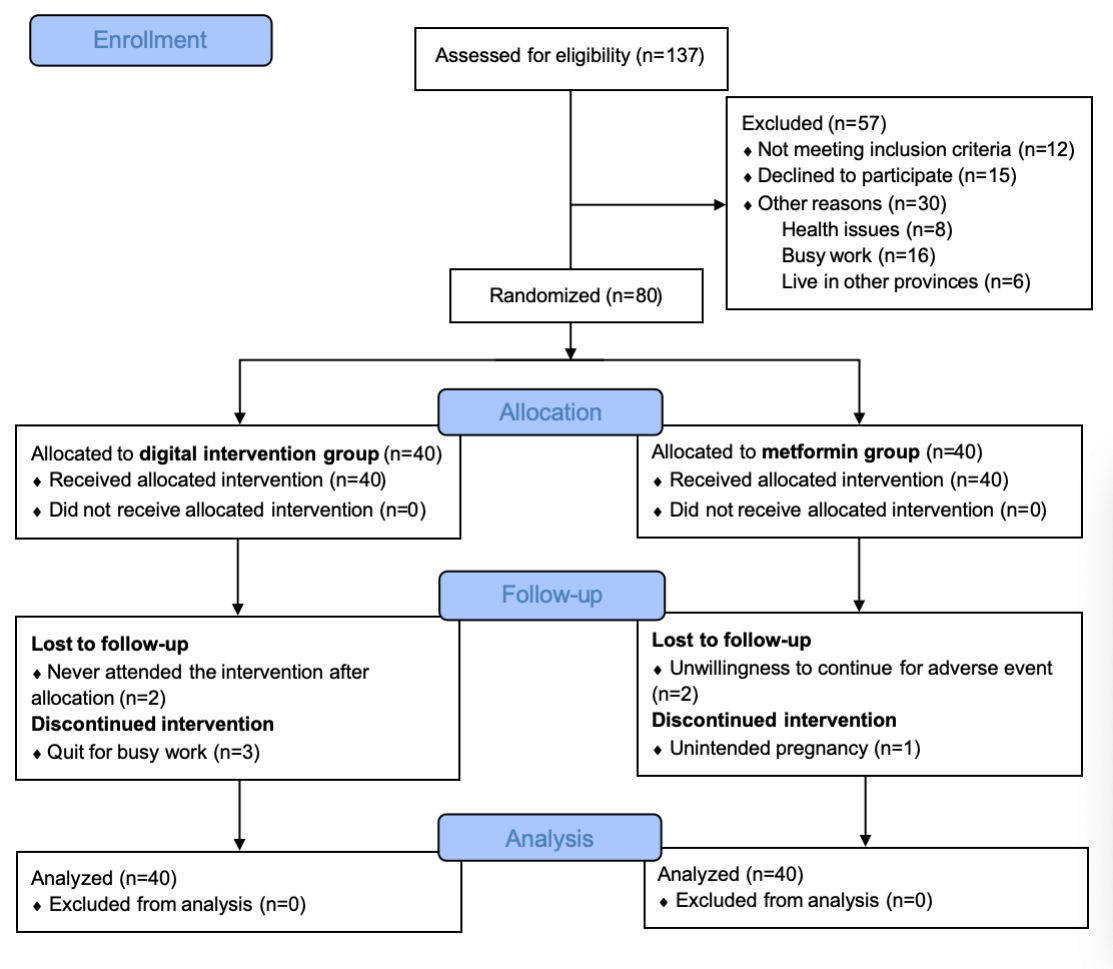
Flow diagram.

The process of recruiting ran from June 2022 to May 2023, and the follow-up assessments were completed in August 2023. Of the 137 outpatients who had PCOS from June 2022 to August 2023, a total of 80 met the admission criteria and volunteered to participate in the study (Figure 2). They were randomly divided into the digital intervention group (40/80, 50%) and metformin group (40/80, 50%). The dropout rates were 13% (5/40) and 8% (3/40) for the digital intervention and metformin groups, respectively. The digital intervention group used a WeChat mini program for 12 weeks, whereas the metformin group received daily doses of 1000 mg of metformin also for 12 weeks. As shown in Multimedia Appendix 4, the adherence rate of the digital intervention group was 89% (95% CI 83%-95%), which was calculated by the participants’ recorded data on the WeChat mini program. On the basis of the number of days participants took metformin within 12 weeks, the adherence rate of the metformin group was 98% (95% CI 96%-99%). The adherence rate was higher in the metformin group than in the digital intervention group (*P*=.003).

Table 1 presents the clinical characteristics measured at baseline. No differences were observed between the two groups in terms of age, body measurements, menstrual cycles, sex hormones, glucose and lipid metabolism, uric acid, body fat distribution, hepatic steatosis, and hepatic fibrosis levels. However, the digital intervention group had a higher mean AST of 25.66 U/L (95% CI 20.78-30.53) compared with the metformin group, which had a mean AST of 19.23 U/L (95% CI 17.00-21.45). Furthermore, there were no appreciable variations in terms of sleep quality, anxiety, depression, eating habits, weekly physical activity, and daily sitting time between the two groups.

**Table 1:** Clinical characteristics of participants measured in the baseline.

Variables	Digital intervention (n=40), mean (95% CI)	Metformin (n=40), mean (95% CI)	*P* value
Age (y)	27.60 (26.22-28.98)	27.95 (26.26-29.64)	.75
Weight (kg)	67.89 (64.00-71.78)	69.23 (65.78-72.68)	.61
BMI (kg/m^2^)	26.22 (24.81-27.63)	26.30 (25.12-27.48)	.93
Waist circumference (cm)	85.63 (82.22-89.03)	84.85 (81.74-87.97)	.74
Hip circumference (cm)	96.57 (94.30-98.84)	98.53 (96.13-100.93)	.23
Waist-to-hip ratio	0.89 (0.86-0.91)	0.86 (0.84-0.88)	.12
Menstrual cycles (number/y)	8.20 (7.20-9.20)	7.82 (6.86-8.78)	.58
Glycated hemoglobin A1c (%)	5.55 (5.43-5.66)	5.54 (5.42-5.66)	.93
Homeostatic model assessment of insulin resistance	4.01 (3.18-4.84)	4.47 (3.54-5.40)	.45
Fasting blood glucose (mmol/L)	4.95 (4.82-5.08)	4.90 (4.70-5.11)	.71
Postprandial blood glucose (mmol/L)	7.60 (6.92-8.27)	7.30 (6.67-7.93)	.52
Fasting insulin (mU/L)	17.82 (14.45-21.20)	19.81 (16.72-22.90)	.38
Postprandial insulin (mU/L)	157.23 (123.22-191.23)	134.35 (103.68-165.01)	.31
Alanine aminotransferase (U/L)	35.63 (25.84-45.43)	26.12 (20.96-31.28)	.09
Aspartate aminotransferase (U/L)	25.66 (20.78-30.53)	19.23 (17.00-21.45)	.*02*^*a*^
Creatinine (μmol/L)	58.02 (54.67-61.37)	57.96 (55.53-60.39)	.98
Uric acid (μmol/L)	372.63 (343.10-402.15)	363.88 (337.28-390.47)	.66
Total cholesterol (mmol/L)	4.98 (4.65-5.31)	4.82 (4.57-5.07)	.45
Triglycerides (mmol/L)	1.45 (1.22-1.68)	1.62 (1.29-1.94)	.39
Low-density lipoprotein cholesterol (mmol/L)	3.22 (2.89-3.54)	2.99 (2.79-3.19)	.24
High-density lipoprotein cholesterol (mmol/L)	1.24 (1.03-1.45)	1.15 (1.06-1.24)	.44
Luteinizing hormone (IU/L)	9.67 (7.61-11.72)	10.33 (7.02-13.64)	.73
Follicle-stimulating hormone (IU/L)	5.43 (4.92-5.94)	5.01 (4.38-5.63)	.30
Total testosterone (ng/mL)	1.83 (1.64-2.02)	1.74 (1.51-1.97)	.54
Free testosterone (pg/mL)	4.42 (3.71-5.13)	4.74 (3.76-5.72)	.59
Androstenedione (ng/mL)	5.68 (4.99-6.37)	5.64 (4.71-6.57)	.95
Dehydroepiandrosterone sulfate (μg/dL)	313.01 (263.32-362.70)	289.74 (245.61-333.87)	.49
Sex hormone–binding globulin (nmol/L)	31.58 (20.92-42.24)	27.58 (22.63-32.53)	.53
Anti-Müllerian hormone (ng/mL)	7.05 (5.59-8.50)	6.14 (5.29-6.99)	.28
Total body fat (%)	43.93 (42.24-45.63)	43.21 (41.73-44.68)	.51
Total body lean mass (%)	52.39 (50.69-54.09)	53.16 (51.67-54.65)	.49
Total fat mass (kg)	29.15 (26.80-31.50)	29.17 (27.23-31.10)	.99
Total lean mass (kg)	34.28 (32.49-36.08)	35.78 (33.81-37.76)	.26
Subcutaneous adipose tissue mass (kg)	1.59 (1.43-1.75)	1.59 (1.45-1.73)	.95
Visceral adipose tissue mass (kg)	0.63 (0.55-0.72)	0.62 (0.56-0.69)	.82
Controlled attenuation parameter (dB/m)	308.68 (291.03-326.32)	316.9 (298.59-335.21)	.52
Liver stiffness measurement (kPa)	5.42 (4.89-5.95)	4.97 (4.62-5.31)	.15
Pittsburgh Sleep Quality Index score	6.35 (5.08-7.02)	6.05 (5.46-7.24)	.30
Hospital Anxiety and Depression–Anxiety Subscale	6.18 (4.82-7.53)	5.13 (3.78-6.47)	.27
Hospital Anxiety and Depression–Depression Subscale	5.60 (4.37-6.83)	5.43 (4.16-6.69)	.18
Uncontrolled eating	20.50 (18.62-22.38)	20.50 (18.70-22.30)	>.99
Cognitive restraint	15.80 (14.72-16.88)	16.13 (14.98-17.28)	.67
Emotional eating	14.38 (12.53-16.22)	14.29 (12.67-15.90)	.94
Total physical activity (metabolic equivalent minutes/wk)	1061.20 (696.61-1425.79)	1538.80 (1003.10-2074.51)	.13
Daily sitting time (min)	465.79 (423.22-508.36)	418.48 (345.91-491.06)	.24

^a^Italicized values indicate *P*<.05.

### Overall Intervention Effect Between Groups

#### Primary Outcome: HOMA-IR

After 12 weeks of treatment, both digital intervention and metformin reduced the HOMA-IR levels (Table 2). The mean change in HOMA-IR from baseline to week 12 was –0.93 (95% CI –1.64 to –0.23) for the digital intervention group and –1.07 (95% CI –2.04 to –.09) for the metformin group. There was no significant difference in HOMA-IR levels between the groups (least squares mean difference –0.20; 95% CI –0.98 to 0.58; *P*=.62), indicating that the improvements in both groups in insulin resistance were comparable.

**Table 2:** Comparison of clinical characteristics between the groups before and after the 12-week study period.

Variables	Digital intervention (n=40), LS^a^ mean (95% CI)	Metformin (n=40), LS mean (95% CI)	Treatment difference (digital intervention–metformin), LS mean (95% CI)	*P* value
Homeostatic model assessment of insulin resistance	–*0.93 (–1.64 to –0.23)*	–*1.07 (–2.04 to –0.09)*	–*0.20 (–0.98 to 0.58)*	*.62* ^*b*^
Weight (kg)	–2.47 (–3.48 to –1.46)	–2.21 (–3.14 to –1.28)	–0.33 (–1.70 to 1.03)	.63
BMI (kg/m^2^)	–0.95 (–1.34 to –0.56)	–0.85 (–1.21 to –0.50)	–0.10 (–0.63 to 0.42)	.69
Waist circumference (cm)	–4.52 (–5.89 to –3.14）	–2.66 (–3.65 to –1.66）	–1.84 (–3.44 to –0.24）	.03
Hip circumference (cm)	–1.98 (–2.87 to –1.09)	–1.80 (–2.45 to –1.15)	–0.29 (–1.37 to 0.79)	.60
Waist-to-hip ratio	–0.03 (–0.04 to –0.02)	–0.01 (–0.02 to 0.00)	–0.02 (–0.03 to 0.00)	.03
Menstrual cycles (no/yr)	1.43 (1.10 to 1.75)	1.51 (1.23 to 1.79)	–0.02 (–0.42 to 0.37)	.91
Glycated hemoglobin A1c (%)	–0.21 (–0.31 to –0.11)	–0.19 (–0.28 to –0.10)	0.00 (–0.11 to 0.11)	.97
Fasting blood glucose (mmol/L)	–0.18 (–0.31 to –0.05)	–0.28 (–0.49 to –0.08)	0.11 (–0.07 to 0.29)	.23
Postprandial blood glucose (mmol/L)	–0.40 (–1.30 to 0.50)	0.02 (–0.68 to 0.71)	–0.03 (–0.90 to 0.85)	.95
Fasting insulin (mU/L)	–3.47 (–6.31 to –0.64)	–3.37 (–6.70 to –0.04)	–1.21 (–4.64 to 2.22)	.48
Postprandial insulin (mU/L)	–59.87 (–106.61 to –13.14)	–7.86 (–37.42 to 21.70)	–33.95 (–76.23 to 8.32)	.11
Alanine aminotransferase (U/L)	–11.36 (–18.89 to –3.83)	–2.81 (–6.37 to 0.76)	–4.34 (–8.75 to 0.08)	.05
Aspartate aminotransferase (U/L)	–5.24 (–10.09 to –0.39)	–1.25 (–3.23 to 0.73)	0.49 (–1.97 to 2.95)	.69
Creatinine (μmol/L)	–1.34 (–4.31 to 1.64)	–2.23 (–4.19 to –0.27)	0.60 (–2.50 to 3.69)	.70
Uric acid (μmol/L)	–9.88 (–34.29 to 14.52)	–3.34 (–21.93 to 15.25)	–3.48 (–29.83 to 22.88)	.79
Total cholesterol (mmol/L)	–0.16 (–0.33 to 0.01)	–0.07 (–0.27 to 0.13)	–0.04 (–0.29 to 0.21)	.74
Triglycerides (mmol/L)	–0.20 (–0.41 to 0.01)	–0.15 (–0.41 to 0.11)	–0.13 (–0.41 to 0.16)	.38
Low-density lipoprotein cholesterol (mmol/L)	–0.13 (–0.29 to 0.04)	0.01 (–0.15 to 0.16)	–0.08 (–0.30 to 0.13)	.44
High-density lipoprotein cholesterol (mmol/L)	–0.14 (–0.38 to 0.09)	–0.01 (–0.07 to 0.05)	–0.00 (–0.10 to 0.09)	.95
Luteinizing hormone (IU/L)	1.72 (–1.49 to 4.94)	–1.65 (–5.17 to 1.86)	3.65 (1.13 to 6.18)	.01
Follicle-stimulating hormone (IU/L)	0.08 (–0.61 to 0.76)	–0.15 (–0.94 to 0.63)	0.43 (–0.42 to 1.28)	.32
Total testosterone (ng/mL)	–0.18 (–0.37 to 0.02)	–0.15 (–0.34 to 0.03)	0.02 (–0.22 to 0.25)	.87
Free testosterone (pg/mL)	s0.24 (–0.99 to 0.51)	–0.77 (–1.44 to –0.10)	0.33 (–0.42 to 1.07)	.38
Androstenedione (ng/mL)	0.11 (–0.89 to 1.12)	0.27 (–0.66 to 1.21)	–0.11 (–1.39 to 1.18)	.87
Dehydroepiandrosterone sulfate (μg/dL)	–17.41 (–55.20 to 20.38)	59.13 (4.34 to 113.92)	–69.73 (–129.70 to –9.75)	.02
Sex hormone–binding globulin (nmol/L)	–1.24 (–11.11 to 8.63)	6.52 (2.44 to 10.60)	–4.61 (–13.81 to 4.59)	.32
Anti-Müllerian hormone (ng/mL)	–0.31 (–1.25 to 0.62)	–0.21 (–0.78 to 0.35)	0.06 (–0.99 to 1.11)	.91
Total body fat (%)	–4.53 (–5.76 to –3.30)	–3.31 (–4.48 to –2.13)	–0.96 (–2.43 to 0.51)	.20
Total body lean mass (%)	4.21 (3.01 to 5.40)	3.22 (2.06 to 4.39)	0.65 (–0.77 to 2.07)	.36
Total fat mass (kg)	–4.44 (–5.60 to –3.29)	–2.86 (–3.66 to –2.05)	–1.59 (–2.88 to –0.30)	.02
Total lean mass (kg)	0.86 (–0.24 to 1.96)	1.02 (0.15 to 1.89)	–0.49 (–1.77 to 0.78)	.44
Subcutaneous adipose tissue mass (kg)	–0.27 (–0.39 to –0.15)	–0.15 (–0.26 to –0.05)	–0.11 (–0.26 to 0.03)	.13
Visceral adipose tissue mass (kg)	–0.12 (–0.18 to –0.06)	–0.09 (–0.13 to –0.05)	–0.02 (–0.09 to 0.04)	.47
Controlled attenuation parameter (dB/m)	–27.00 (–46.02 to –7.98)	–16.75 (–35.27 to 1.77)	–11.34 (–32.18 to 9.51)	.28
Liver stiffness measurement (kPa)	–0.76 (–1.44 to –0.09)	–0.15 (–0.70 to 0.40)	–0.37 (–1.17 to 0.42)	.35
Pittsburgh Sleep Quality Index score	–0.70 (–1.66 to 0.27)	–1.12 (–2.17 to –0.08)	0.40 (–0.93 to 1.72)	.55
Hospital Anxiety and Depression–Anxiety Subscale	–1.33 (–2.24 to –0.43)	–0.55 (–1.52 to 0.43)	–0.53 (–1.75 to 0.68)	.38
Hospital Anxiety and Depression–Depression Subscale	–1.55 (–2.48 to –0.61)	–1.45 (–2.47 to –0.44)	–0.17 (–1.37 to 1.03)	.78
Uncontrolled eating	–2.18 (–3.73 to –0.63)	–2.30 (–3.97 to –0.63)	0.11 (–1.78 to 1.99)	.91
Cognitive restraint	1.88 (0.63 to 3.13)	1.27 (0.08 to 2.46)	0.33 (–1.18 to 1.85)	.66
Emotional eating	–0.91 (–1.80 to –0.02)	–1.42 (–2.26 to –0.59)	0.55 (–0.60 to 1.69)	.35
Total physical activity (metabolic equivalent minutes/wk)	642.69 (176.84 to 1108.54)	–229.23 (–880.15 to 421.69)	373.93 (–218.34 to 966.20)	.21
Daily sitting time (minutes)	–32.81 (–94.76 to 29.13)	17.14 (–37.16 to 71.45)	–26.75 (–94.55 to 41.06)	.43

^a^LS: least squares.

^b^The italicized values are the *P* value of the primary outcome.

#### Secondary Outcomes

After the 12-week intervention, both groups exhibited substantial improvements in body measurements, including body weight, BMI, waist circumference, hip circumference, and waist-to-hip ratio. The digital intervention group exhibited higher improvements in waist circumference (least squares mean difference –1.84 cm; 95% CI –3.44 to –0.24; *P*=.03) and waist-to-hip ratio (least squares mean difference –0.02; 95% CI –0.03 to 0.00; *P*=.03) than the metformin group. In addition, the number of annual menstrual cycles increased in both groups (digital intervention: 1.43, 95% CI 1.10-1.75; metformin: 1.51, 95% CI 1.23-1.79), but the difference was not significant (least squares mean difference –0.02; 95% CI –0.42 to 0.37; *P*=.91).

Considering glucose metabolism, the HbA_1c_, fasting blood glucose, and fasting insulin of both groups significantly improved, and there was no difference in these indices between the two groups. After the 12-week intervention, the lipid metabolism and uric acid levels did not change in either group. After the digital intervention, the ALT, AST, controlled attenuation parameter, and liver stiffness measurement significantly improved; however, no significant difference was observed between the groups. In the metformin group, the creatinine levels decreased, and there was no difference between the groups. In terms of sex hormone–related indicators, the metformin group exhibited a significant decrease in free testosterone levels (–0.77 pg/mL; 95% CI –1.44 to –0.10) but an increase in DHEAS (59.13 μg/dL; 95% CI 4.34-113.92) and SHBG (6.52 nmol/L; 95% CI 2.44-10.60) levels. Although there was no significant change in DHEAS levels in the digital intervention group, there was a decrease compared with the metformin group (least squares mean difference: –69.73 μg/dL, 95% CI –129.70 to –9.75; *P*=.02). In addition, the LH level did not significantly change in either group, but it increased in the digital intervention group compared with the metformin group (least squares mean difference 3.65 IU/L; 95% CI 1.13-6.18; *P*=.005).

After the 12-week intervention, both groups exhibited a notable improvement in body fat distribution. The total body fat, total fat mass, subcutaneous adipose tissue mass, and visceral adipose tissue mass all decreased, whereas the total body lean mass increased in both groups. The digital intervention group exhibited a more significant decrease in total fat mass than the metformin group (least squares mean difference –1.59 kg; 95% CI –2.88 to –0.30; *P*=.02). Furthermore, the metformin group exhibited an increase in total body lean mass, with no difference between the groups.

Digital intervention improved both the HADS-A and HADS-D scores, whereas metformin only improved the HADS-D scores, with no differences between the groups. Both groups exhibited improved eating behaviors, including a decrease in uncontrolled eating, an increase in cognitively restrictive eating, and a decrease in emotional eating, with no differences between the groups. In terms of sleep quality, the mean change in PSQI from baseline to week 12 was –0.70 (–1.66 to 0.27) for the digital intervention group and –1.12 (–2.17 to –0.08) for the metformin group, but there was no difference between the groups (least squares mean difference 0.40; 95% CI –0.93 to 1.72; *P*=.55). The digital intervention effectively increased the weekly total physical activity but did not significantly differ from metformin (least squares mean difference 373.93 MET minutes per week; 95% CI –218.34 to 966.20; *P*=.22).

#### Adverse Events

During the study period, no fatalities or major adverse events were reported (Table 3). In the metformin group, the discontinuation rate due to adverse events was 5% (2/40). The rates of adverse events during the treatment course were 8% (3/40) and 35% (14/40) in the digital intervention and metformin groups, respectively. A minimum of 1 gastrointestinal treatment-emergent adverse event was reported by approximately 30% of the patients in the metformin group. The most frequent gastrointestinal treatment-emergent adverse events were diarrhea (20%), nausea (15%), decreased appetite (8%), and abdominal distension (5%). Most gastrointestinal treatment–emergent adverse events were mild to moderate in severity, with symptoms being noticeable in the first week and then gradually improving over the first month. In the metformin group, severe hypersensitivity reaction and dizziness were reported in one case each. Furthermore, 5% of the participants who received the digital intervention reported feeling hungry and 3% reported feeling dizzy.

**Table 3:** Adverse events and safety data during the treatment in 2 groups.

Event	Digital intervention (n=40), n (%)	Metformin (n=40), n (%)
Gastrointestinal adverse events	0 (0)	12 (30)
Diarrhea	0 (0)	8 (20)
Nausea	0 (0)	6 (15)
Decreased appetite	0 (0)	3 (8)
Abdominal distension	0 (0)	2 (5)
Vomiting	0 (0)	1 (3)
Upper abdominal pain	0 (0)	1 (3)
Constipation	0 (0)	1 (3)
Sensations of hunger	2 (5)	0 (0)
Headache	0 (0)	0 (0)
Hypoglycemia	0 (0)	0 (0)
Severe hypersensitivity reaction	0 (0)	1 (3)
Dizziness	1 (3)	1 (3)

#### Satisfaction With the Program

We also conducted a satisfaction survey for patients with PCOS who completed the 12-week digital intervention (n=35). As presented in Multimedia Appendix 3, in total, 80% of the users reported feeling satisfied overall, 89% of the users thought it saves time, 74% of users thought their PCOS-related issues were resolved, 86% of the users believed that they had a more comprehensive understanding of PCOS, and 80% of the users would recommend the program to others. Of the 35 women who completed the follow-up, 31 responded, with 25 (81%) saying the coaching was most effective, 4 (13%) saying knowledge acquisition was most effective, and 2 (6%) saying tracking was most effective.

## Discussion

### Principal Findings

Our results provide evidence that WeChat-based digital therapies significantly improve self-management and the metabolic and reproductive aspects of the disease among patients with PCOS. While the WeChat-based digital intervention was not statistically different from metformin treatment in terms of lowering HOMA-IR levels, it was more effective in reducing waist circumference, waist-to-hip ratio, total fat mass, and DHEAS. In terms of safety, the main adverse events in the digital intervention group were not as high as those in the metformin group. With a randomized controlled trial design and a 12-week follow-up, the study’s findings offer the first convincing proof of the efficacy and safety of self-supervised, WeChat-based digital intervention to improve PCOS.

Using gold standard procedures, it was estimated that approximately 75% of patients with PCOS had insulin resistance and that the endocrine and reproductive characteristics of PCOS can be brought on by insulin resistance [27]. In clinical studies, HOMA-IR is frequently used as a surrogate marker to detect insulin resistance. Multiple studies have shown that alleviating insulin resistance is closely associated with improving menstrual cycles, ovulation, and in vitro fertilization results in PCOS [28-30]. Consequently, HOMA-IR was selected in this study as the major end point as it plays a significant role in the onset and progression of PCOS. This study demonstrated that, in patients with PCOS with insulin resistance, HOMA-IR can be significantly decreased by digital intervention. Prior research has shown that when several therapy methods (such as metformin, rosiglitazone, and acupuncture) were given for approximately 12 weeks, patients with PCOS experienced improvements in their hyperandrogenemia, weight loss, and metabolic and biochemical parameters [17,25,26].

Furthermore, the study showed that, in the same population, both digital intervention and metformin therapy reduced body weight, BMI, waist circumference, hip circumference, and waist-to-hip ratio; however, the former was associated with a greater reduction in waist circumference and waist-to-hip ratio levels than the latter. Similarly, previous research showed that metformin did not significantly reduce waist circumference as much as lifestyle change did [31,32]. In a recent study, anthropometric indicators, including body weight, waist circumference, and fat mass, significantly improved in the web-based lifestyle intervention group compared with the control group [33]. These results may be attributed to an increase in patients’ physical activities, as only the digital intervention group experienced an increase in weekly total physical activity after the intervention in this study.

Furthermore, the menstrual frequency, HbA_1c_, fasting blood glucose, and fasting insulin of both groups significantly improved; however, there were no significant differences between the groups. Consistent with our results, no significant differences were observed in menstrual patterns between the groups in a previous randomized controlled study of metformin versus lifestyle changes [32]. Another study demonstrated that metformin therapy improved anthropometric parameters, menstrual cycles, and glucose metabolism [34]. Alternatively, the digital intervention significantly reduced the HbA_1c_ levels in patients with prediabetes and diabetes [35,36]. In a study of digital intervention in patients with obesity, the intervention group exhibited significant improvements in HbA_1c_, fasting blood glucose, and HOMA-IR levels while retaining muscle mass and reducing body fat and obtained equivalent outcomes to an intensive obesity management program [37].

The digital intervention group exhibited improved liver function (ALT and AST), liver steatosis, and liver fibrosis but did not show a statistically significant difference from the metformin group. Previous studies have shown that nonalcoholic fatty liver disease and liver function in patients with PCOS could be improved by diet control and exercise [38], and patients in the digital intervention group probably changed their diet and exercise routines because of these benefits in our study. In this study, the creatinine levels also decreased in the metformin group but not in the digital intervention group. Consistently, metformin decreased the serum creatinine levels and renal oxidative damage and fibrosis in a study on diabetic mice [39].

In terms of sex hormones, LH was elevated in the digital intervention group compared with the metformin group, but there was no difference in the LH levels before and after treatment in either group. This may be because not all reproductive hormones in this study were measured during menstruation. As the DHEAS levels increased after metformin treatment, the DHEAS levels decreased in the comparison of differences between the groups (digital intervention–metformin). Metformin therapy has been previously reported to increase DHEAS levels in women with infertility due to PCOS [40]. As metformin can improve insulin sensitivity and adrenal cells have insulin receptors [41], insulin may have an impact on peripheral or adrenal tissue DHEAS metabolism. Meanwhile, the free testosterone levels decreased and the SHBG levels increased in the metformin group. In previous studies, both insulin resistance status improvement and circulating insulin reduction have been shown to increase serum SHBG levels, thereby reducing the blood level of free testosterone in patients with PCOS [34,42].

Because central obesity is frequently associated with patients with PCOS [43], we examined the distribution of body fat in these patients. The percentage of total body fat, percentage of total body lean mass, total fat mass, subcutaneous adipose tissue mass, and visceral adipose tissue mass were improved significantly in both groups, but the decline of the total fat mass was more pronounced in the digital intervention group. This may be explained by the metformin group receiving oral lifestyle guidance. Similar to our results, a meta-analysis suggests that lifestyle interventions are the best way to improve body composition and cardiorespiratory health in women with PCOS [44].

Using the questionnaire, we determined changes in the patients’ sleep quality, mood (anxiety and depression), eating behaviors, and exercise condition. Both groups exhibited significantly improved depression and eating behaviors. The digital intervention improved patients’ anxiety and level of physical activity. In a recent study of digital intervention for other diseases, it effectively improved physical activity, depression, anxiety, and eating behavior [45-47]. Metformin, alternatively, significantly improved sleep quality in our study. Studies have demonstrated that in people with type 2 diabetes, metformin therapy improved sleep quality [48]. In addition, it reduced social stress–related anxiety-like behaviors [49].

In terms of safety, the main adverse events in the digital intervention groups were sensations of hunger (5%) and dizziness (3%). The main adverse events in the metformin groups were gastrointestinal treatment–emergent adverse events (30%). Overall, the digital intervention was found to have fewer side effects, which is advantageous. In the satisfaction questionnaire, most of the patients who completed the follow-up of the digital intervention were satisfied with this treatment and would recommend it to other patients with PCOS. On the basis of the questionnaire’s results, the patients believed that the digital intervention could provide a more thorough understanding of PCOS, solve PCOS-related issues, and save more time. This suggests that the digital intervention is feasible and beneficial while saving doctors’ time and being beneficial for more patients with PCOS. Regarding adherence, while the digital intervention helped patients follow through on lifestyle modifications more successfully, metformin adherence outperformed the digital intervention.

In the digital intervention group, 81% of patients felt that the coaching was most effective. In previous studies, for women with overweight or obesity and PCOS, lifestyle therapies with added SMS text messaging assistance appear to aid in enhancing physical fitness, activity, and aerobic capacity as well as minimize sedentary behavior. It shows that supervision and communication play an important role in maintaining a healthy lifestyle.

### Limitations

However, this study has several limitations. First, the selected population had insulin-resistant PCOS and thus does not represent the entire population. Second, sex hormone indicators were not monitored during menstruation, which affected the judgment of sex hormone results. Third, the participants in this study were not blind, and multiple evaluations of the results increased the chances of type 1 errors. Fourth, no formal evaluation of the cost of the intervention was conducted. Finally, there is a lack of long-term follow-up data to assess whether these changes will persist over time. Thus, multicenter, large-sample studies are warranted to further elucidate the efficacy of digital intervention.

### Conclusions

This study summarizes and confirms the feasibility and application of digital intervention for women with PCOS. The results of the study indicated that the intervention is promising in improving self-management behavior and the metabolic and reproductive features of PCOS. Furthermore, in terms of reducing waist circumference, waist-to-hip ratio, total fat mass, and DHEAS, the digital intervention was more beneficial than metformin. Regarding safety, there were fewer adverse events in the digital intervention group than the metformin group.

## Appendix

Multimedia Appendix 1 CONSORT eHEALTH (Consolidated Standards of Reporting Trials of Electronic and Mobile Health Applications and Online Telehealth) checklist.

Multimedia Appendix 2 The content of 24 videos.

Multimedia Appendix 3 Satisfaction survey on digital intervention (n=35).

Multimedia Appendix 4 The adherence rate of the digital intervention group and metformin group.

## Abbreviations

ALT: alanine aminotransferase
AST: aspartate aminotransferase
CONSORT eHEALTH: Consolidated Standards of Reporting Trials of Electronic and Mobile Health Applications and Online Telehealth
DHEAS: dehydroepiandrosterone sulfate
HADS: Hospital Anxiety and Depression Scale
HbA1c: glycated hemoglobin A1c
HOMA-IR: homeostatic model assessment for insulin resistance
IPAQ: International Physical Activity Questionnaire
LH: luteinizing hormone
MET: metabolic equivalent
PCOS: polycystic ovary syndrome
PSQI: Pittsburgh Sleep Quality Index
SHBG: sex hormone–binding globulin
TFEQ-R21: 21-item Three-Factor Eating Questionnaire
