# Supplementary material for: Efficacy of WeChat-Based Digital Intervention Versus Metformin in Women With Polycystic Ovary Syndrome: Randomized Controlled Trial
Source: J Med Internet Res. 2024 Oct 2;26:e55883. doi: 10.2196/55883 (PMC11483258; doi:10.2196/55883)
Supplement: Multimedia Appendix 1 [file jmir_v26i1e55883_app1.pdf]

CONSORT-EHEALTH (V 1.6.1) - Submission/Publication Form

The CONSORT-EHEALTH checklist is intended for authors of randomized trials evaluating web-based and Internet-based applications/interventions, including mobile interventions, electronic games (incl multiplayer games), social media, certain telehealth applications, and other interactive and/or networked electronic applications. Some of the items (e.g. all subitems under item 5 - description of the intervention) may also be applicable for other study designs.

The goal of the CONSORT EHEALTH checklist and guideline is to be
a) a guide for reporting for authors of RCTs,
b) to form a basis for appraisal of an ehealth trial (in terms of validity)

CONSORT-EHEALTH items/subitems are MANDATORY reporting items for studies published in the Journal of Medical Internet Research and other journals / scientific societies endorsing the checklist.

Items numbered 1., 2., 3., 4a., 4b etc are original CONSORT or CONSORT-NPT (non-pharmacologic treatment) items.
Items with Roman numerals (i., ii, iii, iv etc.) are CONSORT-EHEALTH extensions/clarifications.

As the CONSORT-EHEALTH checklist is still considered in a formative stage, we would ask that you also RATE ON A SCALE OF 1-5 how important/useful you feel each item is FOR THE PURPOSE OF THE CHECKLIST and reporting guideline (optional).

Mandatory reporting items are marked with a red *.
In the textboxes, either copy & paste the relevant sections from your manuscript into this form - please include any quotes from your manuscript in QUOTATION MARKS,
or answer directly by providing additional information not in the manuscript, or elaborating on why the item was not relevant for this study.

YOUR ANSWERS WILL BE PUBLISHED AS A SUPPLEMENTARY FILE TO YOUR PUBLICATION IN JMIR AND ARE CONSIDERED PART OF YOUR PUBLICATION (IF ACCEPTED).
Please fill in these questions diligently. Information will not be copyedited, so please use proper spelling and grammar, use correct capitalization, and avoid abbreviations.

DO NOT FORGET TO SAVE AS PDF _AND_ CLICK THE SUBMIT BUTTON SO YOUR ANSWERS ARE IN OUR DATABASE !!!

Citation Suggestion (if you append the pdf as Appendix we suggest to cite this paper in the caption):

Eysenbach G, CONSORT-EHEALTH Group
CONSORT-EHEALTH: Improving and Standardizing Evaluation Reports of Web-based and Mobile Health Interventions
J Med Internet Res 2011;13(4):e126
URL: http://www.jmir.org/2011/4/e126/
doi: 10.2196/jmir.1923
PMID: 22209829

Sign in to Google to save your progress. Learn more

* Indicates required question

Your name *

First Last

Diliqingna Dilimulati

Primary Affiliation (short), City, Country *

University of Toronto, Toronto, Canada

Tongji university, Shanghai, China

Your e-mail address *

abc@gmail.com

2011337@tongji.edu.cn

Title of your manuscript *

Provide the (draft) title of your manuscript.

Efficacy of WeChat-Based Digital Intervention versus Metformin for Women with Polycystic Ovary Syndrome: Randomized Controlled Trial

Name of your App/Software/Intervention *

If there is a short and a long/alternate name, write the short name first and add the long name in brackets.

PCOS Cloud Classroom

Evaluated Version (if any)

e.g. "V1", "Release 2017-03-01", "Version 2.0.27913"

Your answer

Language(s) *

What language is the intervention/app in? If multiple languages are available, separate by comma (e.g. "English, French")

Chinese

URL of your Intervention Website or App

e.g. a direct link to the mobile app on app in appstore (itunes, Google Play), or URL of the website. If the intervention is a DVD or hardware, you can also link to an Amazon page.

Your answer

URL of an image/screenshot (optional)

Your answer

Accessibility *

Can an enduser access the intervention presently?

- ○ access is free and open
- ○ access only for special usergroups, not open
- ○ access is open to everyone, but requires payment/subscription/in-app purchases
- ◉ app/intervention no longer accessible
- ○ Other:

Primary Medical Indication/Disease/Condition *

e.g. "Stress", "Diabetes", or define the target group in brackets after the condition, e.g. "Autism (Parents of children with)", "Alzheimers (Informal Caregivers of)"

"Polycystic Ovary Syndrome"

Primary Outcomes measured in trial *

comma-separated list of primary outcomes reported in the trial

homeostatic model assessment for insulin res

Secondary/other outcomes

Are there any other outcomes the intervention is expected to affect?

Anthropometric parameters, menstruation frequency, sex hormone levels, metabolic factors, and body fat distribution. Self-assessed online questionnaires on diet, exercise, sleep, anxiety, and depression.

Recommended "Dose" *

What do the instructions for users say on how often the app should be used?

- [x] Approximately Daily
- [ ] Approximately Weekly
- [ ] Approximately Monthly
- [ ] Approximately Yearly
- [ ] "as needed"
- [ ] Other:

Approx. Percentage of Users (starters) still using the app as recommended after 3 months *

- [ ] unknown / not evaluated
- [ ] 0-10%
- [x] 11-20%
- [ ] 21-30%
- [ ] 31-40%
- [ ] 41-50%
- [ ] 51-60%
- [ ] 61-70%
- [ ] 71%-80%
- [ ] 81-90%
- [ ] 91-100%
- [ ] Other:

Overall, was the app/intervention effective? *

- ( ) yes: all primary outcomes were significantly better in intervention group vs control
- (x) partly: SOME primary outcomes were significantly better in intervention group vs control
- ( ) no statistically significant difference between control and intervention
- ( ) potentially harmful: control was significantly better than intervention in one or more outcomes
- ( ) inconclusive: more research is needed
- ( ) Other:

Article Preparation Status/Stage *

At which stage in your article preparation are you currently (at the time you fill in this form)

- ( ) not submitted yet - in early draft status
- ( ) not submitted yet - in late draft status, just before submission
- ( ) submitted to a journal but not reviewed yet
- (x) submitted to a journal and after receiving initial reviewer comments
- ( ) submitted to a journal and accepted, but not published yet
- ( ) published
- ( ) Other:

Journal *

If you already know where you will submit this paper (or if it is already submitted), please provide the journal name (if it is not JMIR, provide the journal name under "other")

- ○ not submitted yet / unclear where I will submit this
- ◉ Journal of Medical Internet Research (JMIR)
- ○ JMIR mHealth and UHealth
- ○ JMIR Serious Games
- ○ JMIR Mental Health
- ○ JMIR Public Health
- ○ JMIR Formative Research
- ○ Other JMIR sister journal
- ○ Other:

Is this a full powered effectiveness trial or a pilot/feasibility trial? *

- ○ Pilot/feasibility
- ◉ Fully powered

Manuscript tracking number *

If this is a JMIR submission, please provide the manuscript tracking number under "other" (The ms tracking number can be found in the submission acknowledgement email, or when you login as author in JMIR. If the paper is already published in JMIR, then the ms tracking number is the four-digit number at the end of the DOI, to be found at the bottom of each published article in JMIR)

- ○ no ms number (yet) / not (yet) submitted to / published in JMIR
- ◉ Other: 55883

TITLE AND ABSTRACT

1a) TITLE: Identification as a randomized trial in the title

1a) Does your paper address CONSORT item 1a? *

I.e does the title contain the phrase "Randomized Controlled Trial"? (if not, explain the reason under "other")

- (•) yes
- ( ) Other:

1a-i) Identify the mode of delivery in the title

Identify the mode of delivery. Preferably use “web-based” and/or “mobile” and/or “electronic game” in the title. Avoid ambiguous terms like “online”, “virtual”, “interactive”. Use “Internet-based” only if Intervention includes non-web-based Internet components (e.g. email), use “computer-based” or “electronic” only if offline products are used. Use “virtual” only in the context of “virtual reality” (3-D worlds). Use “online” only in the context of “online support groups”. Complement or substitute product names with broader terms for the class of products (such as “mobile” or “smart phone” instead of “iphone”), especially if the application runs on different platforms.

	1	2	3	4	5	
subitem not at all important	( )	( )	( )	( )	(•)	essential

Clear selection

Does your paper address subitem 1a-i? *

Copy and paste relevant sections from manuscript title (include quotes in quotation marks "like this" to indicate direct quotes from your manuscript), or elaborate on this item by providing additional information not in the ms, or briefly explain why the item is not applicable/relevant for your study

Yes, it's a "WeChat-Based Digital Intervention".

1a-ii) Non-web-based components or important co-interventions in title

Mention non-web-based components or important co-interventions in title, if any (e.g., “with telephone support”).

	1	2	3	4	5	
subitem not at all important	○	○	●	○	○	essential

Clear selection

Does your paper address subitem 1a-ii?

Copy and paste relevant sections from manuscript title (include quotes in quotation marks "like this" to indicate direct quotes from your manuscript), or elaborate on this item by providing additional information not in the ms, or briefly explain why the item is not applicable/relevant for your study

No, we provided only basic health consolation for participants in both groups, which are not non-web-based components or important co-interventions.

1a-iii) Primary condition or target group in the title

Mention primary condition or target group in the title, if any (e.g., “for children with Type I Diabetes”) Example: A Web-based and Mobile Intervention with Telephone Support for Children with Type I Diabetes: Randomized Controlled Trial

	1	2	3	4	5	
subitem not at all important	○	○	○	○	◉	essential

Clear selection

Does your paper address subitem 1a-iii? *

Copy and paste relevant sections from manuscript title (include quotes in quotation marks "like this" to indicate direct quotes from your manuscript), or elaborate on this item by providing additional information not in the ms, or briefly explain why the item is not applicable/relevant for your study

Yes, the target condition is "Polycystic Ovary Syndrome".

1b) ABSTRACT: Structured summary of trial design, methods, results, and conclusions

NPT extension: Description of experimental treatment, comparator, care providers, centers, and blinding status.

1b-i) Key features/functionalities/components of the intervention and comparator in the METHODS section of the ABSTRACT

Mention key features/functionalities/components of the intervention and comparator in the abstract. If possible, also mention theories and principles used for designing the site. Keep in mind the needs of systematic reviewers and indexers by including important synonyms. (Note: Only report in the abstract what the main paper is reporting. If this information is missing from the main body of text, consider adding it)

	1	2	3	4	5	
subitem not at all important	○	○	○	○	◉	essential

Clear selection

Does your paper address subitem 1b-i? *

Copy and paste relevant sections from the manuscript abstract (include quotes in quotation marks "like this" to indicate direct quotes from your manuscript), or elaborate on this item by providing additional information not in the ms, or briefly explain why the item is not applicable/relevant for your study

Yes. The key intervention is "WeChat-based digital intervention" and "The WeChat-based digital intervention consists of three modules, and a coach will assist the patient in using it." Meanwhile, the comparator is "metformin".

1b-ii) Level of human involvement in the METHODS section of the ABSTRACT

Clarify the level of human involvement in the abstract, e.g., use phrases like “fully automated” vs. “therapist/nurse/care provider/physician-assisted” (mention number and expertise of providers involved, if any). (Note: Only report in the abstract what the main paper is reporting. If this information is missing from the main body of text, consider adding it)

	1	2	3	4	5	
subitem not at all important	○	○	○	○	◉	essential

Clear selection

Does your paper address subitem 1b-ii?

Copy and paste relevant sections from the manuscript abstract (include quotes in quotation marks "like this" to indicate direct quotes from your manuscript), or elaborate on this item by providing additional information not in the ms, or briefly explain why the item is not applicable/relevant for your study

Yes. "The WeChat-based digital intervention consists of three modules, and a coach will assist the patient in using it."

1b-iii) Open vs. closed, web-based (self-assessment) vs. face-to-face assessments in the METHODS section of the ABSTRACT

Mention how participants were recruited (online vs. offline), e.g., from an open access website or from a clinic or a closed online user group (closed usergroup trial), and clarify if this was a purely web-based trial, or there were face-to-face components (as part of the intervention or for assessment). Clearly say if outcomes were self-assessed through questionnaires (as common in web-based trials). Note: In traditional offline trials, an open trial (open-label trial) is a type of clinical trial in which both the researchers and participants know which treatment is being administered. To avoid confusion, use “blinded” or “unblinded” to indicated the level of blinding instead of “open”, as “open” in web-based trials usually refers to “open access” (i.e. participants can self-enrol). (Note: Only report in the abstract what the main paper is reporting. If this information is missing from the main body of text, consider adding it)

	1	2	3	4	5	
subitem not at all important	○	○	○	○	◉	essential

Clear selection

Does your paper address subitem 1b-iii?

Copy and paste relevant sections from the manuscript abstract (include quotes in quotation marks "like this" to indicate direct quotes from your manuscript), or elaborate on this item by providing additional information not in the ms, or briefly explain why the item is not applicable/relevant for your study

Yes. Participants were recruited offline. "A total of 80 women with PCOS and insulin resistance were recruited from an endocrinology clinic" This was not a purely web-based trial. "At baseline and after the 12-week intervention, anthropometric parameters, menstruation frequency, sex hormone levels, metabolic factors, and body fat distribution were obtained at clinic. Besides, self- assessed online questionnaires on diet, exercise, sleep, anxiety, and depression were obtained."

1b-iv) RESULTS section in abstract must contain use data

Report number of participants enrolled/assessed in each group, the use/uptake of the intervention (e.g., attrition/adherence metrics, use over time, number of logins etc.), in addition to primary/secondary outcomes. (Note: Only report in the abstract what the main paper is reporting. If this information is missing from the main body of text, consider adding it)

	1	2	3	4	5	
subitem not at all important	○	○	●	○	○	essential

Clear selection

Does your paper address subitem 1b-iv?

Copy and paste relevant sections from the manuscript abstract (include quotes in quotation marks "like this" to indicate direct quotes from your manuscript), or elaborate on this item by providing additional information not in the ms, or briefly explain why the item is not applicable/relevant for your study

Yes. "A total of 80 women with PCOS and insulin resistance were recruited from an endocrinology clinic and randomly assigned to receive either WeChat-based digital intervention (n = 40) or metformin (n = 40) for 12 weeks." and "A total of 72 participants completed the follow-up (90% follow-up rate), including 35 and 37 from the digital intervention and metformin groups, respectively."

1b-v) CONCLUSIONS/DISCUSSION in abstract for negative trials

Conclusions/Discussions in abstract for negative trials: Discuss the primary outcome - if the trial is negative (primary outcome not changed), and the intervention was not used, discuss whether negative results are attributable to lack of uptake and discuss reasons. (Note: Only report in the abstract what the main paper is reporting. If this information is missing from the main body of text, consider adding it)

	1	2	3	4	5	
subitem not at all important	○	○	●	○	○	essential

Clear selection

Does your paper address subitem 1b-v?

Copy and paste relevant sections from the manuscript abstract (include quotes in quotation marks "like this" to indicate direct quotes from your manuscript), or elaborate on this item by providing additional information not in the ms, or briefly explain why the item is not applicable/relevant for your study

Yes. "The HOMA-IR of the digital intervention group was significantly improved after a 12-week of treatment with a mean change of −0.93 (95% CI, −1.64 to −0.23), but no statistical difference was observed between the groups (least-squares mean difference, −0.20; 95% CI, −0.98 to 0.58; P = .62). Both digital intervention and metformin significantly improved menstruation, and reduced body weight and total fat mass. Furthermore, the digital intervention had a significant advantage over metformin in improving waist circumference, waist–hip ratio, total fat mass, and dehydroepiandrosterone sulfate (DHEAS). In terms of safety, the main adverse events in the digital intervention and metformin groups were sensations of hunger (5%) and gastrointestinal adverse events (30%), respectively."

INTRODUCTION

2a) In INTRODUCTION: Scientific background and explanation of rationale

2a-i) Problem and the type of system/solution

Describe the problem and the type of system/solution that is object of the study: intended as stand-alone intervention vs. incorporated in broader health care program? Intended for a particular patient population? Goals of the intervention, e.g., being more cost-effective to other interventions, replace or complement other solutions? (Note: Details about the intervention are provided in “Methods” under 5)

	1	2	3	4	5	
subitem not at all important	○	○	○	○	◉	essential

Clear selection

Does your paper address subitem 2a-i? *

Copy and paste relevant sections from the manuscript (include quotes in quotation marks "like this" to indicate direct quotes from your manuscript), or elaborate on this item by providing additional information not in the ms, or briefly explain why the item is not applicable/relevant for your study

es.
1) "The first-line treatment for PCOS is lifestyle intervention, and studies have demonstrated that positive lifestyle changes can improve hyperandrogenemia, menstrual cycle, hirsutism, and insulin resistance in the affected patients[5]. However, lifestyle intervention is associated with poor compliance and low sustainability. Nevertheless, there are no indicators to determine whether patients are making enough progress in improving their lifestyle." "Studies have demonstrated that digital therapy accessed on mobile phones, which are convenient and accessible, can effectively improve behavioral changes in diet, exercise, and medication adherence, while predicting disease progression, reducing the frequency of disease-related symptoms, and promoting effective disease management[6, 7]. Regarding PCOS, which is a chronic disease, there are currently limited digital treatment methods."
2) "In this study, we devised and developed a WeChat mini program, which included 24 videos, to help patients with PCOS understand the etiology and pathogenesis of PCOS, which lifestyles can relieve PCOS symptoms, and how to maintain these lifestyles. To achieve self-monitoring, patients need to record their weight, exercise, sleep time, menstruation, and meditation time. Metformin, which improves ovulation and enhances insulin sensitivity, is the first-line insulin-sensitizing medication used to treat PCOS; it was used as a treatment drug in the control group in this study[10]. "
3) The target population of this study is patients with PCOS.
4) "The main purpose of this study was to analyze the effect of digital intervention compared with metformin in improving homeostatic model assessment for insulin resistance (HOMA-IR) levels and other metabolic and reproductive indicators."
"This was a single-center, prospective, randomized controlled clinical trial designed to determine whether digital intervention was effective in patients with PCOS after 12 weeks of treatment. Evaluations were conducted on improvements in PCOS-related clinical parameters and side effects for both groups. Simultaneously, a patient satisfaction survey was conducted for the digital intervention. This study aimed to confirm the efficacy and safety of digital intervention in the treatment of PCOS."

2a-ii) Scientific background, rationale: What is known about the (type of) system

Scientific background, rationale: What is known about the (type of) system that is the object of the study (be sure to discuss the use of similar systems for other conditions/diagnoses, if appropiate), motivation for the study, i.e. what are the reasons for and what is the context for this specific study, from which stakeholder viewpoint is the study performed, potential impact of findings [2]. Briefly justify the choice of the comparator.

	1	2	3	4	5	
subitem not at all important	○	○	○	○	◉	essential

Clear selection

Does your paper address subitem 2a-ii? *

Copy and paste relevant sections from the manuscript (include quotes in quotation marks "like this" to indicate direct quotes from your manuscript), or elaborate on this item by providing additional information not in the ms, or briefly explain why the item is not applicable/relevant for your study

Yes.
1) "A previous three-component lifestyle intervention consisting of diet, exercise, and cognitive behavioral therapy, facilitated by the use of text messaging to track patients' food intake, physical activity, and mood, can effectively improve eating disorder and mood in patients with PCOS and contribute to weight loss."
2) "Studies have demonstrated that digital therapy accessed on mobile phones, which are convenient and accessible, can effectively improve behavioral changes in diet, exercise, and medication adherence, while predicting disease progression, reducing the frequency of disease-related symptoms, and promoting effective disease management. Regarding PCOS, which is a chronic disease, there are currently limited digital treatment methods."
3) "Metformin, which improves ovulation and enhances insulin sensitivity, is the first-line insulin-sensitizing medication used to treat PCOS; it was used as a treatment drug in the control group in this study."

2b) In INTRODUCTION: Specific objectives or hypotheses

Does your paper address CONSORT subitem 2b? *

Copy and paste relevant sections from the manuscript (include quotes in quotation marks "like this" to indicate direct quotes from your manuscript), or elaborate on this item by providing additional information not in the ms, or briefly explain why the item is not applicable/relevant for your study

Yes. "The main purpose of this study was to analyze the effect of digital intervention compared with metformin in improving homeostatic model assessment for insulin resistance (HOMA-IR) levels and other metabolic and reproductive indicators.
This was a single-center, prospective, randomized controlled clinical trial designed to determine whether digital intervention was effective in patients with PCOS after 12 weeks of treatment. Evaluations were conducted on improvements in PCOS-related clinical parameters and side effects for both groups. Simultaneously, a patient satisfaction survey was conducted for the digital intervention. This study aimed to confirm the efficacy and safety of digital intervention in the treatment of PCOS."

METHODS

3a) Description of trial design (such as parallel, factorial) including allocation ratio

Does your paper address CONSORT subitem 3a? *

Copy and paste relevant sections from the manuscript (include quotes in quotation marks "like this" to indicate direct quotes from your manuscript), or elaborate on this item by providing additional information not in the ms, or briefly explain why the item is not applicable/relevant for your study

Yes. "A total of 80 women with PCOS between June 21, 2022, and August 12, 2023, were enrolled in this single-center, 1:1-allocated, unblinded, two-parallel-armed study, from the endocrinology clinic of a tertiary Affiliated Hospital of Tongji University."

3b) Important changes to methods after trial commencement (such as eligibility criteria), with reasons

Does your paper address CONSORT subitem 3b? *

Copy and paste relevant sections from the manuscript (include quotes in quotation marks "like this" to indicate direct quotes from your manuscript), or elaborate on this item by providing additional information not in the ms, or briefly explain why the item is not applicable/relevant for your study

Yes. "No changes were made to this clinical trial after registration."

3b-i) Bug fixes, Downtimes, Content Changes

Bug fixes, Downtimes, Content Changes: ehealth systems are often dynamic systems. A description of changes to methods therefore also includes important changes made on the intervention or comparator during the trial (e.g., major bug fixes or changes in the functionality or content) (5-iii) and other “unexpected events” that may have influenced study design such as staff changes, system failures/downtimes, etc. [2].

	1	2	3	4	5	
subitem not at all important	○	○	○	○	◉	essential

Clear selection

Does your paper address subitem 3b-i?

Copy and paste relevant sections from the manuscript (include quotes in quotation marks "like this" to indicate direct quotes from your manuscript), or elaborate on this item by providing additional information not in the ms, or briefly explain why the item is not applicable/relevant for your study

Yes. "During using the digital intervention, five patients experienced temporary system problems (4 patients had video playback issues and 1 patient had data entry issues). After communicating with the programmer, we optimized and improved the system in time and solved the problems encountered by the patients, but the content has not changed."

4a) Eligibility criteria for participants

Does your paper address CONSORT subitem 4a? *

Copy and paste relevant sections from the manuscript (include quotes in quotation marks "like this" to indicate direct quotes from your manuscript), or elaborate on this item by providing additional information not in the ms, or briefly explain why the item is not applicable/relevant for your study

Yes. "The inclusion criteria were age of 18–45 years, clinical diagnosis of PCOS according to the 2003 Rotterdam diagnostic criteria[11], and patients with HOMA-IR ≥ 1.8 (In Asian countries, HOMA-IR ≥ 1.8 can diagnose insulin resistance and predict the occurrence of metabolic syndrome[12]). Exclusion criteria were: unable to use the WeChat; hyperthyroidism or hypothyroidism; severe abnormal liver function (liver enzyme level more than three times the standard limit); severe abnormal renal function (serum creatinine level > 123.8 μmol/L or estimated glomerular filtration rate < 45 mL·min−1·1.73 m−2); congenital adrenal hyperplasia, hyperprolactinemia, adrenal tumor; accompanied by severe infection, severe anemia, neutropenia and other blood system diseases; type 1 diabetes, monogenic mutation diabetes, or diabetes due to pancreatic damage or other secondary diabetes; existence of mental illness or dementia; history of using contraceptives, metformin, glucagon-like peptide-1 analogues, pioglitazone, all types of antidepressant medications and other drugs in the past 3 months; pregnant or intending to get pregnant within 6 months; participation in another clinical trial within 3 months; declined to participate."

4a-i) Computer / Internet literacy

Computer / Internet literacy is often an implicit “de facto” eligibility criterion - this should be explicitly clarified.

	1	2	3	4	5	
subitem not at all important	○	○	○	●	○	essential

Clear selection

Does your paper address subitem 4a-i?

Copy and paste relevant sections from the manuscript (include quotes in quotation marks "like this" to indicate direct quotes from your manuscript), or elaborate on this item by providing additional information not in the ms, or briefly explain why the item is not applicable/relevant for your study

Yes. "Exclusion criteria were: unable to use the WeChat".

4a-ii) Open vs. closed, web-based vs. face-to-face assessments:

Open vs. closed, web-based vs. face-to-face assessments: Mention how participants were recruited (online vs. offline), e.g., from an open access website or from a clinic, and clarify if this was a purely web-based trial, or there were face-to-face components (as part of the intervention or for assessment), i.e., to what degree got the study team to know the participant. In online-only trials, clarify if participants were quasi-anonymous and whether having multiple identities was possible or whether technical or logistical measures (e.g., cookies, email confirmation, phone calls) were used to detect/prevent these.

	1	2	3	4	5	
subitem not at all important	○	○	○	○	●	essential

Clear selection

Does your paper address subitem 4a-ii? *

Copy and paste relevant sections from the manuscript (include quotes in quotation marks "like this" to indicate direct quotes from your manuscript), or elaborate on this item by providing additional information not in the ms, or briefly explain why the item is not applicable/relevant for your study

Yes. "A total of 80 women with PCOS between June 21, 2022, and August 12, 2023, were enrolled in this single-center, 1:1-allocated, assessor-blinded, two-parallel-armed study, from the endocrinology clinic of a tertiary Affiliated Hospital of Tongji University."
"At baseline and after the 12-week intervention, all clinical and laboratory tests were conducted at clinic with assessors who were blind to the assignment."
"At baseline and after the 12-week intervention, all participants were required to complete the self-assessed online questionnaires, including the 21-item Three-Factor Eating Questionnaire (TFEQ-R21), International Physical Activity Questionnaire (IPAQ), Pittsburgh Sleep Quality Index (PSQI), and Hospital Anxiety and Depression Scale (HADS). Besides, participants in the digital intervention group filled out a self-assessed online satisfaction survey 12 weeks after the intervention, and the five questions in the questionnaire are shown in Multimedia Appendix 3."

4a-iii) Information giving during recruitment

Information given during recruitment. Specify how participants were briefed for recruitment and in the informed consent procedures (e.g., publish the informed consent documentation as appendix, see also item X26), as this information may have an effect on user self-selection, user expectation and may also bias results.

	1	2	3	4	5	
subitem not at all important	○	◉	○	○	○	essential

Clear selection

Does your paper address subitem 4a-iii?

Copy and paste relevant sections from the manuscript (include quotes in quotation marks "like this" to indicate direct quotes from your manuscript), or elaborate on this item by providing additional information not in the ms, or briefly explain why the item is not applicable/relevant for your study

Your answer

4b) Settings and locations where the data were collected

Does your paper address CONSORT subitem 4b? *

Copy and paste relevant sections from the manuscript (include quotes in quotation marks "like this" to indicate direct quotes from your manuscript), or elaborate on this item by providing additional information not in the ms, or briefly explain why the item is not applicable/relevant for your study

Yes. "A total of 80 women with PCOS between June 21, 2022, and August 12, 2023, were enrolled in this single-center, 1:1-allocated, unblinded, two-parallel-armed study, from the endocrinology clinic of a tertiary Affiliated Hospital of Tongji University."

4b-i) Report if outcomes were (self-)assessed through online questionnaires

Clearly report if outcomes were (self-)assessed through online questionnaires (as common in web-based trials) or otherwise.

	1	2	3	4	5	
subitem not at all important	○	○	○	○	◉	essential

Clear selection

Does your paper address subitem 4b-i? *

Copy and paste relevant sections from the manuscript (include quotes in quotation marks "like this" to indicate direct quotes from your manuscript), or elaborate on this item by providing additional information not in the ms, or briefly explain why the item is not applicable/relevant for your study

Yes.
"At baseline and after the 12-week intervention, all participants were required to complete the self-assessed online questionnaires, including the 21-item Three-Factor Eating Questionnaire (TFEQ-R21), International Physical Activity Questionnaire (IPAQ), Pittsburgh Sleep Quality Index (PSQI), and Hospital Anxiety and Depression Scale (HADS). Besides, participants in the digital intervention group filled out a self-assessed online satisfaction survey 12 weeks after the intervention, and the five questions in the questionnaire are shown in Multimedia Appendix 3."

4b-ii) Report how institutional affiliations are displayed

Report how institutional affiliations are displayed to potential participants [on ehealth media], as affiliations with prestigious hospitals or universities may affect volunteer rates, use, and reactions with regards to an intervention.(Not a required item – describe only if this may bias results)

	1	2	3	4	5	
subitem not at all important	◉	○	○	○	○	essential

Clear selection

Does your paper address subitem 4b-ii?

Copy and paste relevant sections from the manuscript (include quotes in quotation marks "like this" to indicate direct quotes from your manuscript), or elaborate on this item by providing additional information not in the ms, or briefly explain why the item is not applicable/relevant for your study

Your answer

5) The interventions for each group with sufficient details to allow replication, including how and when they were actually administered

5-i) Mention names, credential, affiliations of the developers, sponsors, and owners

Mention names, credential, affiliations of the developers, sponsors, and owners [6] (if authors/evaluators are owners or developer of the software, this needs to be declared in a “Conflict of interest” section or mentioned elsewhere in the manuscript).

	1	2	3	4	5	
subitem not at all important	(●)	( )	( )	( )	( )	essential

Clear selection

Does your paper address subitem 5-i?

Copy and paste relevant sections from the manuscript (include quotes in quotation marks "like this" to indicate direct quotes from your manuscript), or elaborate on this item by providing additional information not in the ms, or briefly explain why the item is not applicable/relevant for your study

Your answer

5-ii) Describe the history/development process

Describe the history/development process of the application and previous formative evaluations (e.g., focus groups, usability testing), as these will have an impact on adoption/use rates and help with interpreting results.

	1	2	3	4	5	
subitem not at all important	(●)	( )	( )	( )	( )	essential

Clear selection

Does your paper address subitem 5-ii?

Copy and paste relevant sections from the manuscript (include quotes in quotation marks "like this" to indicate direct quotes from your manuscript), or elaborate on this item by providing additional information not in the ms, or briefly explain why the item is not applicable/relevant for your study

Your answer

5-iii) Revisions and updating

Revisions and updating. Clearly mention the date and/or version number of the application/intervention (and comparator, if applicable) evaluated, or describe whether the intervention underwent major changes during the evaluation process, or whether the development and/or content was “frozen” during the trial. Describe dynamic components such as news feeds or changing content which may have an impact on the replicability of the intervention (for unexpected events see item 3b).

	1	2	3	4	5	
subitem not at all important	(•)	( )	( )	( )	( )	essential

Clear selection

Does your paper address subitem 5-iii?

Copy and paste relevant sections from the manuscript (include quotes in quotation marks "like this" to indicate direct quotes from your manuscript), or elaborate on this item by providing additional information not in the ms, or briefly explain why the item is not applicable/relevant for your study

Your answer

5-iv) Quality assurance methods

Provide information on quality assurance methods to ensure accuracy and quality of information provided [1], if applicable.

	1	2	3	4	5	
subitem not at all important	◉	○	○	○	○	essential

Clear selection

Does your paper address subitem 5-iv?

Copy and paste relevant sections from the manuscript (include quotes in quotation marks "like this" to indicate direct quotes from your manuscript), or elaborate on this item by providing additional information not in the ms, or briefly explain why the item is not applicable/relevant for your study

Your answer

5-v) Ensure replicability by publishing the source code, and/or providing screenshots/screen-capture video, and/or providing flowcharts of the algorithms used

Ensure replicability by publishing the source code, and/or providing screenshots/screen-capture video, and/or providing flowcharts of the algorithms used. Replicability (i.e., other researchers should in principle be able to replicate the study) is a hallmark of scientific reporting.

	1	2	3	4	5	
subitem not at all important	◉	○	○	○	○	essential

Clear selection

Does your paper address subitem 5-v?

Copy and paste relevant sections from the manuscript (include quotes in quotation marks "like this" to indicate direct quotes from your manuscript), or elaborate on this item by providing additional information not in the ms, or briefly explain why the item is not applicable/relevant for your study

Your answer

5-vi) Digital preservation

Digital preservation: Provide the URL of the application, but as the intervention is likely to change or disappear over the course of the years; also make sure the intervention is archived (Internet Archive, webcitation.org, and/or publishing the source code or screenshots/videos alongside the article). As pages behind login screens cannot be archived, consider creating demo pages which are accessible without login.

	1	2	3	4	5	
subitem not at all important	(•)	( )	( )	( )	( )	essential

Clear selection

Does your paper address subitem 5-vi?

Copy and paste relevant sections from the manuscript (include quotes in quotation marks "like this" to indicate direct quotes from your manuscript), or elaborate on this item by providing additional information not in the ms, or briefly explain why the item is not applicable/relevant for your study

Your answer

5-vii) Access

Access: Describe how participants accessed the application, in what setting/context, if they had to pay (or were paid) or not, whether they had to be a member of specific group. If known, describe how participants obtained “access to the platform and Internet” [1]. To ensure access for editors/reviewers/readers, consider to provide a “backdoor” login account or demo mode for reviewers/readers to explore the application (also important for archiving purposes, see vi).

	1	2	3	4	5	
subitem not at all important	○	○	◉	○	○	essential

Clear selection

Does your paper address subitem 5-vii? *

Copy and paste relevant sections from the manuscript (include quotes in quotation marks "like this" to indicate direct quotes from your manuscript), or elaborate on this item by providing additional information not in the ms, or briefly explain why the item is not applicable/relevant for your study

Yes. "PCOS patients assigned to the digital intervention group can register a new account for free through WeChat, and they need to use the WeChat mini program for 12 weeks."

5-viii) Mode of delivery, features/functionalities/components of the intervention and comparator, and the theoretical framework

Describe mode of delivery, features/functionalities/components of the intervention and comparator, and the theoretical framework [6] used to design them (instructional strategy [1], behaviour change techniques, persuasive features, etc., see e.g., [7, 8] for terminology). This includes an in-depth description of the content (including where it is coming from and who developed it) [1],” whether [and how] it is tailored to individual circumstances and allows users to track their progress and receive feedback” [6]. This also includes a description of communication delivery channels and – if computer-mediated communication is a component – whether communication was synchronous or asynchronous [6]. It also includes information on presentation strategies [1], including page design principles, average amount of text on pages, presence of hyperlinks to other resources, etc. [1].

	1	2	3	4	5	
subitem not at all important	○	○	○	○	◉	essential

Clear selection

Does your paper address subitem 5-viii? *

Copy and paste relevant sections from the manuscript (include quotes in quotation marks "like this" to indicate direct quotes from your manuscript), or elaborate on this item by providing additional information not in the ms, or briefly explain why the item is not applicable/relevant for your study

Yes. "Digital intervention group
The WeChat mini program was designed by Endocrinology and Metabolism Department of Shanghai Tenth People's Hospital and developed by Shanghai Zoey Information Technology Co., LTD. PCOS patients assigned to the digital intervention group can register a new account for free through WeChat, and they need to use the WeChat mini program for 12 weeks. As presented in Figure 1, the program consists of three modules. In module 1, patients can watch two videos per week, with each video being about 5 min long; a total of 24 videos can be watched over a 12-week period. These videos describe the four elements of a healthy lifestyle that should be prioritized by patients with PCOS: healthy diet, regular exercise, good sleep, and stress reduction. The video specifically explains how the four elements affect the occurrence and development of PCOS, what can be effectively done to achieve a good lifestyle, and how to adhere to these lifestyles. Multimedia Appendix 1 presents the title and main content of each video. Patients need to record their menstruation (duration of menstruation, amount of bleeding, body temperature, and mood), weight (weight change value, body mass index [BMI], and weight status appearing after weight submission), exercise time (according to different exercise intensities, low-, moderate-, and high-intensity exercises were recorded, and the type of exercise needed to be selected), sleep time (bedtime and wake-up time), and meditation time (also provides 24 pieces of meditation music) every day. Simultaneously, the list of recommended food and food to avoid is provided in the food recommendation part. After recording, the patients can see a statistical chart of their weight and exercise in module 2. To allow patients to sufficiently understand the important role of lifestyle improvement in PCOS treatment, we have set up module 3, which they can selectively read by themselves, including an overall introduction to PCOS, healthy lifestyles suitable for PCOS, different dietary patterns (including low-carb, high-protein, and restricted energy-balanced diets), choices of different exercises (the intensity of each exercise and the amount of energy that can be consumed), and importance of sleep hygiene (the dangers of circadian disruption). Meanwhile, the doctor acts as the patient's coach, downloading the patient's record data every week; analyzing the patient's performance that week; summarizing the weekly exercise time, weight, sleep, and menstrual period and sending it to the patient; and suggesting possible improvements. In addition, the doctor asks the patients if they encounter problems that cannot be solved and provides support and encouragement. During using the digital intervention, five patients experienced temporary system problems (4 patients had video playback issues and 1 patient had data entry issues). After communicating with the programmer, we optimized and improved the system in time and solved the problems encountered by the patients, but the content has not changed."
"Metformin group
During the 12-week intervention, the metformin group was administered 1000 mg of metformin (Glucophage, Merck KGaA) per day. In previous clinical trials, the dose of metformin ranged from 850 mg to 2000 mg/day[13]. To reduce the side effects of drugs, the dose of 1000 mg/day was selected for this study, and a doctor educated the patients regarding healthy lifestyles during the first visit. "

5-ix) Describe use parameters

Describe use parameters (e.g., intended “doses” and optimal timing for use). Clarify what instructions or recommendations were given to the user, e.g., regarding timing, frequency, heaviness of use, if any, or was the intervention used ad libitum.

	1	2	3	4	5	
subitem not at all important	○	○	○	○	●	essential

Clear selection

Does your paper address subitem 5-ix?

Copy and paste relevant sections from the manuscript (include quotes in quotation marks "like this" to indicate direct quotes from your manuscript), or elaborate on this item by providing additional information not in the ms, or briefly explain why the item is not applicable/relevant for your study

Yes. "PCOS patients assigned to the digital intervention group can register a new account for free through WeChat, and they need to use the WeChat mini program for 12 weeks." "As presented in Figure 1, the program consists of three modules. In module 1, patients can watch two videos per week, with each video being about 5 min long; a total of 24 videos can be watched over a 12-week period." "need to record their menstruation during their menstrual period (duration of menstruation, amount of bleeding, body temperature, and mood), and record their daily weight (weight change value, body mass index [BMI], and weight status appearing after weight submission), daily exercise time (according to different exercise intensities, low-, moderate-, and high-intensity exercises were recorded, and the type of exercise needed to be selected), daily sleep time (bedtime and wake-up time), and daily meditation time (also provides 24 pieces of meditation music)." "Meanwhile, the doctor acts as the patient’s coach, downloading the patient’s record data every week; analyzing the patient’s performance that week; summarizing the weekly exercise time, weight, sleep, and menstrual period and sending it to the patient; and suggesting possible improvements."

5-x) Clarify the level of human involvement

Clarify the level of human involvement (care providers or health professionals, also technical assistance) in the e-intervention or as co-intervention (detail number and expertise of professionals involved, if any, as well as “type of assistance offered, the timing and frequency of the support, how it is initiated, and the medium by which the assistance is delivered”. It may be necessary to distinguish between the level of human involvement required for the trial, and the level of human involvement required for a routine application outside of a RCT setting (discuss under item 21 – generalizability).

	1	2	3	4	5	
subitem not at all important	○	○	○	◉	○	essential

Clear selection

Does your paper address subitem 5-x?

Copy and paste relevant sections from the manuscript (include quotes in quotation marks "like this" to indicate direct quotes from your manuscript), or elaborate on this item by providing additional information not in the ms, or briefly explain why the item is not applicable/relevant for your study

Yes. "Meanwhile, the doctor acts as the patient’s coach, downloading the patient’s record data every week; analyzing the patient’s performance that week; summarizing the weekly exercise time, weight, sleep, and menstrual period and sending it to the patient; and suggesting possible improvements."

5-xi) Report any prompts/reminders used

Report any prompts/reminders used: Clarify if there were prompts (letters, emails, phone calls, SMS) to use the application, what triggered them, frequency etc. It may be necessary to distinguish between the level of prompts/reminders required for the trial, and the level of prompts/reminders for a routine application outside of a RCT setting (discuss under item 21 – generalizability).

	1	2	3	4	5	
subitem not at all important	○	○	○	○	◉	essential

Clear selection

Does your paper address subitem 5-xi? *

Copy and paste relevant sections from the manuscript (include quotes in quotation marks "like this" to indicate direct quotes from your manuscript), or elaborate on this item by providing additional information not in the ms, or briefly explain why the item is not applicable/relevant for your study

Yes. "Meanwhile, the doctor acts as the patient’s coach, downloading the patient’s record data every week; analyzing the patient’s performance that week; summarizing the weekly exercise time, weight, sleep, and menstrual period and sending it to the patient; and suggesting possible improvements."

5-xii) Describe any co-interventions (incl. training/support)

Describe any co-interventions (incl. training/support): Clearly state any interventions that are provided in addition to the targeted eHealth intervention, as ehealth intervention may not be designed as stand-alone intervention. This includes training sessions and support [1]. It may be necessary to distinguish between the level of training required for the trial, and the level of training for a routine application outside of a RCT setting (discuss under item 21 – generalizability.

	1	2	3	4	5	
subitem not at all important	○	○	○	○	◉	essential

Clear selection

Does your paper address subitem 5-xii? *

Copy and paste relevant sections from the manuscript (include quotes in quotation marks "like this" to indicate direct quotes from your manuscript), or elaborate on this item by providing additional information not in the ms, or briefly explain why the item is not applicable/relevant for your study

Yes. Digital intervention group: "Meanwhile, the doctor acts as the patient’s coach, downloading the patient’s record data every week; analyzing the patient’s performance that week; summarizing the weekly exercise time, weight, sleep, and menstrual period and sending it to the patient; and suggesting possible improvements."
Metformin group: "a doctor educated the patients regarding healthy lifestyles during the first visit."

6a) Completely defined pre-specified primary and secondary outcome measures, including how and when they were assessed

Does your paper address CONSORT subitem 6a? *

Copy and paste relevant sections from the manuscript (include quotes in quotation marks "like this" to indicate direct quotes from your manuscript), or elaborate on this item by providing additional information not in the ms, or briefly explain why the item is not applicable/relevant for your study

Yes.
Clinical and laboratory measurements
The primary outcome was HOMA-IR alterations, whereas the secondary outcomes were changes in menstrual cycles, metabolic indicators, sex hormones, body composition, and degree of fibrosis and steatosis in the liver. Furthermore, body weight, waist circumference, and hip circumference were collected. Body weight (kg)/height (m2) was used to determine the BMI and waist circumference (cm)/hip circumference (cm) was used to determine the waist–hip ratio. Menstrual cycle is defined as the total number of menstruations in the past 12 months. The following metabolic variables were assessed: fasting blood glucose, fasting insulin, total cholesterol, triglycerides, high-density lipoprotein cholesterol (HDL-c), low-density lipoprotein cholesterol (LDL-c), alanine aminotransferase (ALT), aspartate aminotransferase (AST), creatinine, uric acid, sex hormone-binding globulin (SHBG) and glycated hemoglobin A1c (HbA1c). A 75-g oral glucose tolerance test was employed to evaluate the postprandial blood glucose and postprandial insulin levels at 120 min. Sex hormones included the luteinizing hormone (LH), follicle-stimulating hormone (FSH), total testosterone, free testosterone, androstenedione, anti-Mullerian hormone and dehydroepiandrosterone sulfate (DHEAS). (fasting blood glucose (mmol/L) × fasting insulin (mU/L))/22.5 was used to calculate the HOMA-IR[13]. Transient elastography (Echosens, Paris) was employed to measure the controlled attenuation parameter (CAP) and determine the liver stiffness measurement (LSM), which represent the extents of liver steatosis and fibrosis, respectively. Dual-energy X-ray absorptiometry (Hologic, Marlborough) was used to measure body composition. At baseline and after the 12-week intervention, all clinical and laboratory tests were conducted at clinic.
Questionnaires
At baseline and after the 12-week intervention, all participants were required to complete the self-assessed online questionnaires, including the 21-item Three-Factor Eating Questionnaire (TFEQ-R21), International Physical Activity Questionnaire (IPAQ), Pittsburgh Sleep Quality Index (PSQI), and Hospital Anxiety and Depression Scale (HADS). Besides, participants in the digital intervention group filled out a self-assessed online satisfaction survey 12 weeks after the intervention, and the five questions in the questionnaire are shown in Multimedia Appendix 3.
TFEQ-R21 is a questionnaire used to examine eating behavior[14]. The three scales comprising the TFEQ-R21 are the cognitive restraint scale (6 items), uncontrolled eating scale (9 items), and emotional eating scale (6 items). These scales cover different aspects of eating behavior. The higher the score, the more severe the behavior of cognitive restraint, uncontrolled eating, or emotional eating.
The translated short form of the IPAQ was employed to self-evaluate physical activity, and there was a 7-day recall period. The usefulness of the IPAQ has been validated. It provides data on the amount of time spent each week on several physical activity domains, including low, moderate, and vigorous intensity[15, 16]. Metabolic equivalent (MET) is a measure of energy expenditure expressed as a multiple of resting energy costs and can be used to quantify the intensity of different activities. By calculating MET × days × daily time, standardized techniques were employed to convert time, days per activity, and intensity to MET minute/week scores. One minute of walking, 1 min of general moderate-intensity activity, and 1 min of vigorous exercise were equivalent to 3.3, 4.0, and 8.0 METs, respectively.
The PSQI is a self-report assessment tool that measures sleep quality over a 30-day period[17]. The scale yields a global score and seven component scores, namely, subjective

[illegible] sleep quality, sleep latency, duration, efficiency, sleep disruptions, use of sleeping pills, and dysfunction throughout the day. Every element receives a score between 0 and 3, and the overall result can range from 0 to 21. A higher number indicates lower sleep quality.
A 14-item test called the HADS is used to assess an individual's anxiety and depression levels[18]. Seven of the items are associated with depression and the remaining items with anxiety. The four response possibilities on each item range from 0 to 3, enabling the subscales of anxiety and depression (HADS-A and HADS-D) to have independent total scores, each ranging from 0 to 21. While the scores of 0–7 are considered as "normal" and 8–10 as "borderline", scores of 11 or more on each subscale are considered to indicate serious instances of psychological morbidity.

6a-i) Online questionnaires: describe if they were validated for online use and apply CHERRIES items to describe how the questionnaires were designed/deployed

If outcomes were obtained through online questionnaires, describe if they were validated for online use and apply CHERRIES items to describe how the questionnaires were designed/deployed [9].

	1	2	3	4	5	
subitem not at all important	○	◉	○	○	○	essential

Clear selection

Does your paper address subitem 6a-i?

Copy and paste relevant sections from manuscript text

Your answer

6a-ii) Describe whether and how “use” (including intensity of use/dosage) was defined/measured/monitored

Describe whether and how “use” (including intensity of use/dosage) was defined/measured/monitored (logins, logfile analysis, etc.). Use/adoption metrics are important process outcomes that should be reported in any ehealth trial.

	1	2	3	4	5	
subitem not at all important	○	○	◉	○	○	essential

Clear selection

Does your paper address subitem 6a-ii?

Copy and paste relevant sections from manuscript text

Yes. "In module 1, patients can watch two videos per week, with each video being about 5 min long; a total of 24 videos can be watched over a 12-week period. These videos describe the four elements of a healthy lifestyle that should be prioritized by patients with PCOS: healthy diet, regular exercise, good sleep, and stress reduction. The video specifically explains how the four elements affect the occurrence and development of PCOS, what can be effectively done to achieve a good lifestyle, and how to adhere to these lifestyles. Multimedia Appendix 1 presents the title and main content of each video. In module 2, patients need to record the patients' their menstruation during their menstrual period (duration of menstruation, amount of bleeding, body temperature, and mood), and record their daily weight (weight change value, body mass index [BMI], and weight status appearing after weight submission), daily exercise time (according to different exercise intensities, low-, moderate-, and high-intensity exercises were recorded, and the type of exercise needed to be selected), daily sleep time (bedtime and wake-up time), and daily meditation time (also provides 24 pieces of meditation music). Simultaneously, the list of recommended food and food to avoid is provided in the food recommendation part. After recording, the patients can see a statistical chart of their weight and exercise in module 2. To allow patients to sufficiently understand the important role of lifestyle improvement in PCOS treatment, we have set up module 3, which they can selectively read by themselves, including an overall introduction to PCOS, healthy lifestyles suitable for PCOS, different dietary patterns (including low-carb, high-protein, and restricted energy-balanced diets), choices of different exercises (the intensity of each exercise and the amount of energy that can be consumed), and importance of sleep hygiene (the dangers of circadian disruption). Meanwhile, the doctor acts as the patient's coach, downloading the patient's record data every week; analyzing the patient's performance that week; summarizing the weekly exercise time, weight, sleep, and menstrual period and sending it to the patient; and suggesting possible improvements. In addition, the doctor asks the patients if they encounter problems that cannot be solved and provides support and encouragement."

6a-iii) Describe whether, how, and when qualitative feedback from participants was obtained

Describe whether, how, and when qualitative feedback from participants was obtained (e.g., through emails, feedback forms, interviews, focus groups).

	1	2	3	4	5	
subitem not at all important	○	○	○	◉	○	essential

Clear selection

Does your paper address subitem 6a-iii?

Copy and paste relevant sections from manuscript text

Yes. "Meanwhile, the doctor acts as the patient’s coach, downloading the patient’s record data every week; analyzing the patient’s performance that week; summarizing the weekly exercise time, weight, sleep, and menstrual period and sending it to the patient; and suggesting possible improvements."

6b) Any changes to trial outcomes after the trial commenced, with reasons

Does your paper address CONSORT subitem 6b? *

Copy and paste relevant sections from the manuscript (include quotes in quotation marks "like this" to indicate direct quotes from your manuscript), or elaborate on this item by providing additional information not in the ms, or briefly explain why the item is not applicable/relevant for your study

Yes. "No changes were made to this clinical trial after registration."

7a) How sample size was determined

NPT: When applicable, details of whether and how the clustering by care provides or centers was addressed

7a-i) Describe whether and how expected attrition was taken into account when calculating the sample size

Describe whether and how expected attrition was taken into account when calculating the sample size.

	1	2	3	4	5	
subitem not at all important	○	○	○	○	◉	essential

Clear selection

Does your paper address subitem 7a-i?

Copy and paste relevant sections from manuscript title (include quotes in quotation marks "like this" to indicate direct quotes from your manuscript), or elaborate on this item by providing additional information not in the ms, or briefly explain why the item is not applicable/relevant for your study

Yes. "Based on previous research[19-21], we calculated the mean difference in the HOMA-IR levels between pre- and post-metformin intervention as 1.0 and the standard deviation of the outcome variable as 1.0. Alternatively, we predicted the mean difference in the HOMA-IR levels between pre- and post-digital intervention to be 2.0 and the standard deviation of the outcome variable to be 1.0. Our power analysis revealed that according to an online calculator (MedSci Sample Size tools), with a 30% sample loss rate, a minimum of 30 participants were required for each group to achieve a significant outcome (alpha = .05, power = .90); the total sample size of the study was 60."

7b) When applicable, explanation of any interim analyses and stopping guidelines

Does your paper address CONSORT subitem 7b? *

Copy and paste relevant sections from the manuscript (include quotes in quotation marks "like this" to indicate direct quotes from your manuscript), or elaborate on this item by providing additional information not in the ms, or briefly explain why the item is not applicable/relevant for your study

No. No interim analysis was performed in this study.

8a) Method used to generate the random allocation sequence

NPT: When applicable, how care providers were allocated to each trial group

Does your paper address CONSORT subitem 8a? *

Copy and paste relevant sections from the manuscript (include quotes in quotation marks "like this" to indicate direct quotes from your manuscript), or elaborate on this item by providing additional information not in the ms, or briefly explain why the item is not applicable/relevant for your study

Yes. This trial used a simple randomization approach. "Following baseline evaluation, based on a predefined computer-generated number in a 1:1 assignment, the participants were randomly assigned to receive either digital intervention or metformin"

8b) Type of randomisation; details of any restriction (such as blocking and block size)

Does your paper address CONSORT subitem 8b? *

Copy and paste relevant sections from the manuscript (include quotes in quotation marks "like this" to indicate direct quotes from your manuscript), or elaborate on this item by providing additional information not in the ms, or briefly explain why the item is not applicable/relevant for your study

Yes. This trial used a simple randomization approach. "Following baseline evaluation, based on a predefined computer-generated number in a 1:1 assignment, the participants were randomly assigned to receive either digital intervention or metformin."

9) Mechanism used to implement the random allocation sequence (such as sequentially numbered containers), describing any steps taken to conceal the sequence until interventions were assigned

Does your paper address CONSORT subitem 9? *

Copy and paste relevant sections from the manuscript (include quotes in quotation marks "like this" to indicate direct quotes from your manuscript), or elaborate on this item by providing additional information not in the ms, or briefly explain why the item is not applicable/relevant for your study

Yes. This trial used a simple randomization approach. "Following baseline evaluation, based on a predefined computer-generated number in a 1:1 assignment, the participants were randomly assigned to receive either digital intervention or metformin. One researcher created the random number sequence for this investigation, and another researcher enrolled and assigned participants.

10) Who generated the random allocation sequence, who enrolled participants, and who assigned participants to interventions

Does your paper address CONSORT subitem 10? *

Copy and paste relevant sections from the manuscript (include quotes in quotation marks "like this" to indicate direct quotes from your manuscript), or elaborate on this item by providing additional information not in the ms, or briefly explain why the item is not applicable/relevant for your study

Yes. "One researcher created the random number sequence for this investigation, and another researcher enrolled and assigned participants."

11a) If done, who was blinded after assignment to interventions (for example, participants, care providers, those assessing outcomes) and how

NPT: Whether or not administering co-interventions were blinded to group assignment

11a-i) Specify who was blinded, and who wasn't

Specify who was blinded, and who wasn't. Usually, in web-based trials it is not possible to blind the participants [1, 3] (this should be clearly acknowledged), but it may be possible to blind outcome assessors, those doing data analysis or those administering co-interventions (if any).

	1	2	3	4	5	
subitem not at all important	○	○	○	○	◉	essential

Clear selection

Does your paper address subitem 11a-i? *

Copy and paste relevant sections from the manuscript (include quotes in quotation marks "like this" to indicate direct quotes from your manuscript), or elaborate on this item by providing additional information not in the ms, or briefly explain why the item is not applicable/relevant for your study

Yes. Assessors, participants and care providers were all unblinded.

11a-ii) Discuss e.g., whether participants knew which intervention was the “intervention of interest” and which one was the “comparator”

Informed consent procedures (4a-ii) can create biases and certain expectations - discuss e.g., whether participants knew which intervention was the “intervention of interest” and which one was the “comparator”.

	1	2	3	4	5	
subitem not at all important	○	○	○	◉	○	essential

Clear selection

Does your paper address subitem 11a-ii?

Copy and paste relevant sections from the manuscript (include quotes in quotation marks "like this" to indicate direct quotes from your manuscript), or elaborate on this item by providing additional information not in the ms, or briefly explain why the item is not applicable/relevant for your study

Yes. "During intervention, both groups were aware of their allocation."

11b) If relevant, description of the similarity of interventions

(this item is usually not relevant for ehealth trials as it refers to similarity of a placebo or sham intervention to a active medication/intervention)

Does your paper address CONSORT subitem 11b? *

Copy and paste relevant sections from the manuscript (include quotes in quotation marks "like this" to indicate direct quotes from your manuscript), or elaborate on this item by providing additional information not in the ms, or briefly explain why the item is not applicable/relevant for your study

No. In this study, one group was digital intervention and the other group was drug intervention, which did not involve similarity.

12a) Statistical methods used to compare groups for primary and secondary outcomes

NPT: When applicable, details of whether and how the clustering by care providers or centers was addressed

Does your paper address CONSORT subitem 12a? *

Copy and paste relevant sections from the manuscript (include quotes in quotation marks "like this" to indicate direct quotes from your manuscript), or elaborate on this item by providing additional information not in the ms, or briefly explain why the item is not applicable/relevant for your study

Yes. "The intention-to-treat principle was employed for all data analyses, and SPSS version 25.0 was used for all statistical analyses. Continuous variables were expressed as means with 95% confidence intervals (CIs). Mean differences between the two treatment groups are displayed as least-squares mean changes and 95% CIs. To evaluate the group differences in the primary and continuous secondary endpoints, we employed an analysis of covariance model in which the treatment was a fixed effect and the corresponding baseline value was a covariate. Fisher's exact test or chi-squared test was used where applicable to analyze categorical variables. $P < 0.05$ was considered to indicate statistical significance."

12a-i) Imputation techniques to deal with attrition / missing values

Imputation techniques to deal with attrition / missing values: Not all participants will use the intervention/comparator as intended and attrition is typically high in ehealth trials. Specify how participants who did not use the application or dropped out from the trial were treated in the statistical analysis (a complete case analysis is strongly discouraged, and simple imputation techniques such as LOCF may also be problematic [4]).

	1	2	3	4	5	
subitem not at all important	○	○	●	○	○	essential

Clear selection

Does your paper address subitem 12a-i? *

Copy and paste relevant sections from the manuscript (include quotes in quotation marks "like this" to indicate direct quotes from your manuscript), or elaborate on this item by providing additional information not in the ms, or briefly explain why the item is not applicable/relevant for your study

Yes. "The intention-to-treat principle was employed for all data analyses" and "To evaluate the group differences in the primary and continuous secondary endpoints, we employed an analysis of covariance model in which the treatment was a fixed effect and the corresponding baseline value was a covariate." Missing values were not processed.

12b) Methods for additional analyses, such as subgroup analyses and adjusted analyses

Does your paper address CONSORT subitem 12b? *

Copy and paste relevant sections from the manuscript (include quotes in quotation marks "like this" to indicate direct quotes from your manuscript), or elaborate on this item by providing additional information not in the ms, or briefly explain why the item is not applicable/relevant for your study

No. There is no any other analysis performed.

X26) REB/IRB Approval and Ethical Considerations [recommended as subheading under "Methods"] (not a CONSORT item)

X26-i) Comment on ethics committee approval

	1	2	3	4	5	
subitem not at all important	○	○	○	◉	○	essential

Clear selection

Does your paper address subitem X26-i?

Copy and paste relevant sections from the manuscript (include quotes in quotation marks "like this" to indicate direct quotes from your manuscript), or elaborate on this item by providing additional information not in the ms, or briefly explain why the item is not applicable/relevant for your study

Yes. "The study protocol was approved by the ethics committee of the Shanghai Tenth People's Hospital (SHSY-IEC-5.0/21K191/P02)."

x26-ii) Outline informed consent procedures

Outline informed consent procedures e.g., if consent was obtained offline or online (how? Checkbox, etc.?), and what information was provided (see 4a-ii). See [6] for some items to be included in informed consent documents.

	1	2	3	4	5	
subitem not at all important	○	◉	○	○	○	essential

Clear selection

Does your paper address subitem X26-ii?

Copy and paste relevant sections from the manuscript (include quotes in quotation marks "like this" to indicate direct quotes from your manuscript), or elaborate on this item by providing additional information not in the ms, or briefly explain why the item is not applicable/relevant for your study

Your answer

X26-iii) Safety and security procedures

Safety and security procedures, incl. privacy considerations, and any steps taken to reduce the likelihood or detection of harm (e.g., education and training, availability of a hotline)

	1	2	3	4	5	
subitem not at all important	○	◉	○	○	○	essential

Clear selection

Does your paper address subitem X26-iii?

Copy and paste relevant sections from the manuscript (include quotes in quotation marks "like this" to indicate direct quotes from your manuscript), or elaborate on this item by providing additional information not in the ms, or briefly explain why the item is not applicable/relevant for your study

Your answer

RESULTS

13a) For each group, the numbers of participants who were randomly assigned, received intended treatment, and were analysed for the primary outcome

NPT: The number of care providers or centers performing the intervention in each group and the number of patients treated by each care provider in each center

Does your paper address CONSORT subitem 13a? *

Copy and paste relevant sections from the manuscript (include quotes in quotation marks "like this" to indicate direct quotes from your manuscript), or elaborate on this item by providing additional information not in the ms, or briefly explain why the item is not applicable/relevant for your study

Yes. "Of the 137 outpatients who had PCOS, 80 met the admission criteria and volunteered to participate in the study (Figure 2). They were randomly divided into the digital intervention group (n = 40) and metformin group (n = 40). The dropout rates were 13% (5/40) and 8% (3/40) for the digital intervention and metformin groups, respectively. "

13b) For each group, losses and exclusions after randomisation, together with reasons

Does your paper address CONSORT subitem 13b? (NOTE: Preferably, this is shown in a CONSORT flow diagram) *

Copy and paste relevant sections from the manuscript (include quotes in quotation marks "like this" to indicate direct quotes from your manuscript), or elaborate on this item by providing additional information not in the ms, or briefly explain why the item is not applicable/relevant for your study

Yes. This is shown in flow chart.

13b-i) Attrition diagram

Strongly recommended: An attrition diagram (e.g., proportion of participants still logging in or using the intervention/comparator in each group plotted over time, similar to a survival curve) or other figures or tables demonstrating usage/dose/engagement.

	1	2	3	4	5	
subitem not at all important	○	○	○	○	◉	essential

Clear selection

Does your paper address subitem 13b-i?

Copy and paste relevant sections from the manuscript or cite the figure number if applicable (include quotes in quotation marks "like this" to indicate direct quotes from your manuscript), or elaborate on this item by providing additional information not in the ms, or briefly explain why the item is not applicable/relevant for your study

Yes. "As showed in Multimedia Appendix 2, the adherence rate of the digital intervention group was 89% (95% CI from 83 to 95), which was calculated by the participants' recorded data on the WeChat mini program. Based on the number of days participants took metformin within 12 weeks, the adherence rate of the metformin group was 98% (95% CI from 96 to 99). The adherence rate was higher in the metformin group than in the digital intervention group (P = .003)."

14a) Dates defining the periods of recruitment and follow-up

Does your paper address CONSORT subitem 14a? *

Copy and paste relevant sections from the manuscript (include quotes in quotation marks "like this" to indicate direct quotes from your manuscript), or elaborate on this item by providing additional information not in the ms, or briefly explain why the item is not applicable/relevant for your study

Yes. "The process of recruiting ran from June 2022 to May 2023, and the follow-up assessments were completed in August 2023."

14a-i) Indicate if critical "secular events" fell into the study period

Indicate if critical "secular events" fell into the study period, e.g., significant changes in Internet resources available or "changes in computer hardware or Internet delivery resources"

	1	2	3	4	5	
subitem not at all important	○	◉	○	○	○	essential

Clear selection

Does your paper address subitem 14a-i?

Copy and paste relevant sections from the manuscript (include quotes in quotation marks "like this" to indicate direct quotes from your manuscript), or elaborate on this item by providing additional information not in the ms, or briefly explain why the item is not applicable/relevant for your study

Your answer

14b) Why the trial ended or was stopped (early)

Does your paper address CONSORT subitem 14b? *

Copy and paste relevant sections from the manuscript (include quotes in quotation marks "like this" to indicate direct quotes from your manuscript), or elaborate on this item by providing additional information not in the ms, or briefly explain why the item is not applicable/relevant for your study

No. The trial did not stop halfway.

15) A table showing baseline demographic and clinical characteristics for each group

NPT: When applicable, a description of care providers (case volume, qualification, expertise, etc.) and centers (volume) in each group

Does your paper address CONSORT subitem 15? *

Copy and paste relevant sections from the manuscript (include quotes in quotation marks "like this" to indicate direct quotes from your manuscript), or elaborate on this item by providing additional information not in the ms, or briefly explain why the item is not applicable/relevant for your study

Yes. These information was shown in Table 1.

15-i) Report demographics associated with digital divide issues

In ehealth trials it is particularly important to report demographics associated with digital divide issues, such as age, education, gender, social-economic status, computer/Internet/ehealth literacy of the participants, if known.

	1	2	3	4	5	
subitem not at all important	○	○	○	●	○	essential

Clear selection

Does your paper address subitem 15-i? *

Copy and paste relevant sections from the manuscript (include quotes in quotation marks "like this" to indicate direct quotes from your manuscript), or elaborate on this item by providing additional information not in the ms, or briefly explain why the item is not applicable/relevant for your study

Yes. Age status was reported in Table 1 and all participants are female.

16) For each group, number of participants (denominator) included in each analysis and whether the analysis was by original assigned groups

16-i) Report multiple "denominators" and provide definitions

Report multiple "denominators" and provide definitions: Report N's (and effect sizes) "across a range of study participation [and use] thresholds" [1], e.g., N exposed, N consented, N used more than x times, N used more than y weeks, N participants "used" the intervention/comparator at specific pre-defined time points of interest (in absolute and relative numbers per group). Always clearly define "use" of the intervention.

	1	2	3	4	5	
subitem not at all important	○	○	○	○	●	essential

Clear selection

Does your paper address subitem 16-i? *

Copy and paste relevant sections from the manuscript (include quotes in quotation marks "like this" to indicate direct quotes from your manuscript), or elaborate on this item by providing additional information not in the ms, or briefly explain why the item is not applicable/relevant for your study

Yes. "As showed in Multimedia Appendix 2, the adherence rate of the digital intervention group was 89% (95% CI from 83 to 95), which was calculated by the participants' recorded data on the WeChat mini program. Based on the number of days participants took metformin within 12 weeks, the adherence rate of the metformin group was 98% (95% CI from 96 to 99). The adherence rate was higher in the metformin group than in the digital intervention group (P = .003)."

16-ii) Primary analysis should be intent-to-treat

Primary analysis should be intent-to-treat, secondary analyses could include comparing only "users", with the appropriate caveats that this is no longer a randomized sample (see 18-i).

	1	2	3	4	5	
subitem not at all important	○	○	○	○	◉	essential

Clear selection

Does your paper address subitem 16-ii?

Copy and paste relevant sections from the manuscript (include quotes in quotation marks "like this" to indicate direct quotes from your manuscript), or elaborate on this item by providing additional information not in the ms, or briefly explain why the item is not applicable/relevant for your study

Yes. "The intention-to-treat principle was employed for all data analyses".

17a) For each primary and secondary outcome, results for each group, and the estimated effect size and its precision (such as 95% confidence interval)

Does your paper address CONSORT subitem 17a? *

Copy and paste relevant sections from the manuscript (include quotes in quotation marks "like this" to indicate direct quotes from your manuscript), or elaborate on this item by providing additional information not in the ms, or briefly explain why the item is not applicable/relevant for your study

Yes. "After 12 weeks of treatment, both digital intervention and metformin reduced the HOMA-IR levels (Table 2). The mean change in HOMA-IR from baseline to week 12 was −0.93 (95% CI, −1.64 to −0.23) for the digital intervention group and −1.07 (95% CI −2.04 to −0.09) for the metformin group. There was no significant difference in HOMA-IR between the groups (least-squares mean difference, −0.20; 95% CI, −0.98 to 0.58; P = .62), indicating that the improvements in both groups in insulin resistance were comparable. After the 12-week intervention, both groups exhibited significant improvements in body measurements, including body weight, BMI, waist circumference, hip circumference, and waist–hip ratio. The digital intervention group exhibited higher improvements in waist circumference (least-squares mean difference, −1.84 cm; 95% CI, −3.44 to −0.24; P = .03) and waist–hip ratio (least-squares mean difference, −0.02; 95% CI, −0.03 to 0.00; P = .03) than the metformin group. In addition, the number of annual menstrual cycles increased in both groups (digital intervention: 1.43, 95% CI, 1.10 to 1.75; metformin: 1.51, 95% CI, 1.23 to 1.79), but the difference was not significant (least-squares mean difference, −0.02; 95% CI, −0.42 to 0.37; P = .91)."

17a-i) Presentation of process outcomes such as metrics of use and intensity of use

In addition to primary/secondary (clinical) outcomes, the presentation of process outcomes such as metrics of use and intensity of use (dose, exposure) and their operational definitions is critical. This does not only refer to metrics of attrition (13-b) (often a binary variable), but also to more continuous exposure metrics such as “average session length”. These must be accompanied by a technical description how a metric like a “session” is defined (e.g., timeout after idle time) [1] (report under item 6a).

	1	2	3	4	5	
subitem not at all important	○	○	●	○	○	essential

Clear selection

Does your paper address subitem 17a-i?

Copy and paste relevant sections from the manuscript (include quotes in quotation marks "like this" to indicate direct quotes from your manuscript), or elaborate on this item by providing additional information not in the ms, or briefly explain why the item is not applicable/relevant for your study

Your answer

17b) For binary outcomes, presentation of both absolute and relative effect sizes is recommended

Does your paper address CONSORT subitem 17b? *

Copy and paste relevant sections from the manuscript (include quotes in quotation marks "like this" to indicate direct quotes from your manuscript), or elaborate on this item by providing additional information not in the ms, or briefly explain why the item is not applicable/relevant for your study

Yes. "During the study period, no fatalities or major adverse events were reported (Table 3). In the metformin group, the discontinuation rate due to adverse events was 5% (2/40). The rates of adverse events during the treatment course were 8% (3/40) and 35% (14/40) in the digital intervention and metformin groups, respectively. A minimum of one gastrointestinal treatment-emergent adverse events was reported by about 30% of the patients in the metformin group. The most frequent gastrointestinal treatment-emergent adverse events were diarrhea (20%), nausea (15%), decreased appetite (8%), and abdominal distension (5%). Most gastrointestinal treatment-emergent adverse events were mild to moderate in severity, with symptoms being noticeable in the first week and then gradually improving over the first month. In the metformin group, severe hypersensitivity reaction and dizziness were reported in one case each. Furthermore, 5% of the participants who received the digital intervention reported feeling hungry and 3% reported feeling dizzy."

18) Results of any other analyses performed, including subgroup analyses and adjusted analyses, distinguishing pre-specified from exploratory

Does your paper address CONSORT subitem 18? *

Copy and paste relevant sections from the manuscript (include quotes in quotation marks "like this" to indicate direct quotes from your manuscript), or elaborate on this item by providing additional information not in the ms, or briefly explain why the item is not applicable/relevant for your study

No other analysis was performed.

18-i) Subgroup analysis of comparing only users

A subgroup analysis of comparing only users is not uncommon in ehealth trials, but if done, it must be stressed that this is a self-selected sample and no longer an unbiased sample from a randomized trial (see 16-iii).

	1	2	3	4	5	
subitem not at all important	○	○	○	◉	○	essential

Clear selection

Does your paper address subitem 18-i?

Copy and paste relevant sections from the manuscript (include quotes in quotation marks "like this" to indicate direct quotes from your manuscript), or elaborate on this item by providing additional information not in the ms, or briefly explain why the item is not applicable/relevant for your study

Yes. "We also conducted a satisfaction survey for patients with PCOS who completed the 12-week digital intervention (n = 35). As presented in Multimedia Appendix 3, 80% of the users reported feeling satisfied overall, 89% of the users thought it saves time, 74% of users thought their PCOS-related issues were resolved, 86% of the users believed that they had a more comprehensive understanding of PCOS, and 80% of the users would recommend the program to others."

19) All important harms or unintended effects in each group

(for specific guidance see CONSORT for harms)

Does your paper address CONSORT subitem 19? *

Copy and paste relevant sections from the manuscript (include quotes in quotation marks "like this" to indicate direct quotes from your manuscript), or elaborate on this item by providing additional information not in the ms, or briefly explain why the item is not applicable/relevant for your study

Yes. "During the study period, no fatalities or major adverse events were reported (Table 3). In the metformin group, the discontinuation rate due to adverse events was 5% (2/40). The rates of adverse events during the treatment course were 8% (3/40) and 35% (14/40) in the digital intervention and metformin groups, respectively. A minimum of one gastrointestinal treatment-emergent adverse events was reported by about 30% of the patients in the metformin group. The most frequent gastrointestinal treatment-emergent adverse events were diarrhea (20%), nausea (15%), decreased appetite (8%), and abdominal distension (5%). Most gastrointestinal treatment-emergent adverse events were mild to moderate in severity, with symptoms being noticeable in the first week and then gradually improving over the first month. In the metformin group, severe hypersensitivity reaction and dizziness were reported in one case each. Furthermore, 5% of the participants who received the digital intervention reported feeling hungry and 3% reported feeling dizzy."

19-i) Include privacy breaches, technical problems

Include privacy breaches, technical problems. This does not only include physical “harm” to participants, but also incidents such as perceived or real privacy breaches [1], technical problems, and other unexpected/unintended incidents. “Unintended effects” also includes unintended positive effects [2].

	1	2	3	4	5	
subitem not at all important	○	◉	○	○	○	essential

Clear selection

Does your paper address subitem 19-i?

Copy and paste relevant sections from the manuscript (include quotes in quotation marks "like this" to indicate direct quotes from your manuscript), or elaborate on this item by providing additional information not in the ms, or briefly explain why the item is not applicable/relevant for your study

Your answer

19-ii) Include qualitative feedback from participants or observations from staff/researchers

Include qualitative feedback from participants or observations from staff/researchers, if available, on strengths and shortcomings of the application, especially if they point to unintended/unexpected effects or uses. This includes (if available) reasons for why people did or did not use the application as intended by the developers.

	1	2	3	4	5	
subitem not at all important	○	◉	○	○	○	essential

Clear selection

Does your paper address subitem 19-ii?

Copy and paste relevant sections from the manuscript (include quotes in quotation marks "like this" to indicate direct quotes from your manuscript), or elaborate on this item by providing additional information not in the ms, or briefly explain why the item is not applicable/relevant for your study

Your answer

DISCUSSION

22) Interpretation consistent with results, balancing benefits and harms, and considering other relevant evidence

NPT: In addition, take into account the choice of the comparator, lack of or partial blinding, and unequal expertise of care providers or centers in each group

22-i) Restate study questions and summarize the answers suggested by the data, starting with primary outcomes and process outcomes (use)

Restate study questions and summarize the answers suggested by the data, starting with primary outcomes and process outcomes (use).

	1	2	3	4	5	
subitem not at all important	○	○	○	◉	○	essential

Clear selection

Does your paper address subitem 22-i? *

Copy and paste relevant sections from the manuscript (include quotes in quotation marks "like this" to indicate direct quotes from your manuscript), or elaborate on this item by providing additional information not in the ms, or briefly explain why the item is not applicable/relevant for your study

Yes. "Our results provide evidence that WeChat-based digital therapies significantly improve PCOS patients' self-management, and the metabolic and reproductive aspects of the disease. While WeChat-based digital intervention was not statistically different from metformin treatment in terms of lowering HOMA-IR levels, it was more effective in reducing waist circumference, waist-to-hip ratio, total fat mass, and DHEAS. With an RCT design and a 12-week follow-up, the study's findings offer the first convincing proof of the efficacy of a self-supervised, WeChat-based digital intervention to improve PCOS."

22-ii) Highlight unanswered new questions, suggest future research

Highlight unanswered new questions, suggest future research.

	1	2	3	4	5	
subitem not at all important	○	○	○	◉	○	essential

Clear selection

Does your paper address subitem 22-ii?

Copy and paste relevant sections from the manuscript (include quotes in quotation marks "like this" to indicate direct quotes from your manuscript), or elaborate on this item by providing additional information not in the ms, or briefly explain why the item is not applicable/relevant for your study

Yes. "However, this study has several limitations. First, the selected population had insulin resistant and thus does not represent the entire population. Second, sex hormone indicators were not monitored during menstruation, which affected the judgment of sex hormone results. Third, the participants in this study were not blind and multiple evaluations of the results increased the chances of type I errors. Thus, multi- center, large-sample studies are warranted to further elucidate the efficacy of digital intervention."

20) Trial limitations, addressing sources of potential bias, imprecision, and, if relevant, multiplicity of analyses

20-i) Typical limitations in ehealth trials

Typical limitations in ehealth trials: Participants in ehealth trials are rarely blinded. Ehealth trials often look at a multiplicity of outcomes, increasing risk for a Type I error. Discuss biases due to non-use of the intervention/usability issues, biases through informed consent procedures, unexpected events.

	1	2	3	4	5	
subitem not at all important	○	○	○	◉	○	essential

Clear selection

Does your paper address subitem 20-i? *

Copy and paste relevant sections from the manuscript (include quotes in quotation marks "like this" to indicate direct quotes from your manuscript), or elaborate on this item by providing additional information not in the ms, or briefly explain why the item is not applicable/relevant for your study

Yes. "Third, the participants in this study were not blind and multiple evaluations of the results increased the chances of type I errors."

21) Generalisability (external validity, applicability) of the trial findings

NPT: External validity of the trial findings according to the intervention, comparators, patients, and care providers or centers involved in the trial

21-i) Generalizability to other populations

Generalizability to other populations: In particular, discuss generalizability to a general Internet population, outside of a RCT setting, and general patient population, including applicability of the study results for other organizations

	1	2	3	4	5	
subitem not at all important	○	◉	○	○	○	essential

Clear selection

Does your paper address subitem 21-i?

Copy and paste relevant sections from the manuscript (include quotes in quotation marks "like this" to indicate direct quotes from your manuscript), or elaborate on this item by providing additional information not in the ms, or briefly explain why the item is not applicable/relevant for your study

Your answer

21-ii) Discuss if there were elements in the RCT that would be different in a routine application setting

Discuss if there were elements in the RCT that would be different in a routine application setting (e.g., prompts/reminders, more human involvement, training sessions or other co-interventions) and what impact the omission of these elements could have on use, adoption, or outcomes if the intervention is applied outside of a RCT setting.

	1	2	3	4	5	
subitem not at all important	○	◉	○	○	○	essential

Clear selection

Does your paper address subitem 21-ii?

Copy and paste relevant sections from the manuscript (include quotes in quotation marks "like this" to indicate direct quotes from your manuscript), or elaborate on this item by providing additional information not in the ms, or briefly explain why the item is not applicable/relevant for your study

Your answer

OTHER INFORMATION

23) Registration number and name of trial registry

Does your paper address CONSORT subitem 23? *

Copy and paste relevant sections from the manuscript (include quotes in quotation marks "like this" to indicate direct quotes from your manuscript), or elaborate on this item by providing additional information not in the ms, or briefly explain why the item is not applicable/relevant for your study

Yes. 'The study was registered in ClinicalTrials.gov (NCT05386706), and the name of trial registry is “Digital Intervention PCOS”.'

24) Where the full trial protocol can be accessed, if available

Does your paper address CONSORT subitem 24? *

Cite a Multimedia Appendix, other reference, or copy and paste relevant sections from the manuscript (include quotes in quotation marks "like this" to indicate direct quotes from your manuscript), or elaborate on this item by providing additional information not in the ms, or briefly explain why the item is not applicable/relevant for your study

No. The protocol of this study was not published.

25) Sources of funding and other support (such as supply of drugs), role of funders

Does your paper address CONSORT subitem 25? *

Copy and paste relevant sections from the manuscript (include quotes in quotation marks "like this" to indicate direct quotes from your manuscript), or elaborate on this item by providing additional information not in the ms, or briefly explain why the item is not applicable/relevant for your study

Yes. "This research has been supported by funding from the Shanghai Tenth People's Hospital Funding for clinical research projects YNCR2C013, the National Nature Science Foundation of China (Nos. 81601269, 82170861, 82103713 and 81970677), and the SHDC Clinical Research Plan (No. SHDC2020CR1017B)."

X27) Conflicts of Interest (not a CONSORT item)

X27-i) State the relation of the study team towards the system being evaluated

In addition to the usual declaration of interests (financial or otherwise), also state the relation of the study team towards the system being evaluated, i.e., state if the authors/evaluators are distinct from or identical with the developers/sponsors of the intervention.

	1	2	3	4	5	
subitem not at all important	○	○	○	○	●	essential

Clear selection

Does your paper address subitem X27-i?

Copy and paste relevant sections from the manuscript (include quotes in quotation marks "like this" to indicate direct quotes from your manuscript), or elaborate on this item by providing additional information not in the ms, or briefly explain why the item is not applicable/relevant for your study

Yes. "The authors declare no competing interests."

About the CONSORT EHEALTH checklist

As a result of using this checklist, did you make changes in your manuscript? *

- ● yes, major changes
- ○ yes, minor changes
- ○ no

What were the most important changes you made as a result of using this checklist?

To add more details related to the application and the implementation process.

How much time did you spend on going through the checklist INCLUDING making changes in your manuscript *

It took me about seven hours.

As a result of using this checklist, do you think your manuscript has improved? *

- (●) yes
- ( ) no
- ( ) Other:

Would you like to become involved in the CONSORT EHEALTH group?

This would involve for example becoming involved in participating in a workshop and writing an "Explanation and Elaboration" document

- (●) yes
- ( ) no
- ( ) Other:

Clear selection

Any other comments or questions on CONSORT EHEALTH

I have no further comments or questions.

STOP - Save this form as PDF before you click submit

To generate a record that you filled in this form, we recommend to generate a PDF of this page (on a Mac, simply select "print" and then select "print as PDF") before you submit it.

When you submit your (revised) paper to JMIR, please upload the PDF as supplementary file.

Don't worry if some text in the textboxes is cut off, as we still have the complete information in our database. Thank you!

Final step: Click submit !

Click submit so we have your answers in our database!

Submit Clear form

Never submit passwords through Google Forms.

This content is neither created nor endorsed by Google. Report Abuse - Terms of Service - Privacy Policy

Google Forms
