# Supplementary material for: Efficacy of WeChat-Based Digital Intervention Versus Metformin in Women With Polycystic Ovary Syndrome: Randomized Controlled Trial
Source: J Med Internet Res. 2024 Oct 2;26:e55883. doi: 10.2196/55883 (PMC11483258; doi:10.2196/55883)
Supplement: Multimedia Appendix 2 [file jmir_v26i1e55883_app2.docx]

**Multimedia Appendix 2: The content of 24 videos.**

| **Time** | **Video title** | **Video content** |
| --- | --- | --- |
| Week 1 | 1. Can lifestyle intervention improve PCOS？ | Introduce the lifestyle changes that patients with PCOS need to make, and how to use this WeChat mini-program. |
|  | 2. You need a comprehensive understanding of PCOS. | The symptoms, morbidity, potential risks and treatment of PCOS are introduced. |
| Week 2 | 3. Why do you suffer from PCOS, and how should you face it? | The pathogenesis of PCOS includes genetic and environmental factors, of which environmental factors can be improved by lifestyle changes. |
|  | 1. How to cure PCOS effectively? | Develop good eating habits, integrate exercise into daily life, don't stay up late, rationally arrange sleep time, and manage emotions and stress. |
| Week 3 | 5. How do PCOS girls eat healthy? | Choose natural, low glycemic index, anti-inflammatory, antioxidant foods, as well as good carbohydrates and good fats. |
|  | 6. What are the best foods for PCOS girls to eat? | Introduce some low glycemic index, anti-inflammatory, antioxidant foods, and good carbohydrates and fats. |
| Week 4 | 7. What sports are recommended? | Recommended exercises include jumping rope, swimming, fast walking, cycling, jogging and so on. |
|  | 8. How do you find time to exercise? | Run early in the morning, walk to and from work, take advantage of leisure breaks, and set specific goals. |
| Week 5 | 9. What is essential to improve PCOS? | Sleep is crucial for improving PCOS. Good sleep requires regularity and a good atmosphere before going to bed. |
|  | 10. How to adjust sleep time? | Introduce the dangers of staying up late, and some ways to regulate sleep time. |
| Week 6 | 11. Stress and bad moods can worsen PCOS. | The influence of stress and bad mood on PCOS. The active treatment of anxiety and depression can improve PCOS. |
|  | 12. Are there any tips to relieve bad emotions in life? | Methods of mood regulation. |
| Week 7 | 13. Why are obese girls more likely to develop PCOS? | Obesity can lead to metabolic disorders that trigger PCOS. Relationship between obesity and PCOS. |
|  | 14. What should PCOS girls with acne pay attention to? | Avoid high-sugar diets, dairy products, staying up late, learn to reduce mental stress, and take omega-3, vitamin A and zinc supplements. |
| Week 8 | 15. Can you recover menstruation without drugs? | Taking hormonal drugs is not ideal for treating PCOS, and fostering a healthy lifestyle can fundamentally improve PCOS. |
|  | 16. Why can some hypoglycemic drugs treat PCOS? | Hypoglycemic drugs can improve insulin resistance, and also reduce body weight and body fat percentage. |
| Week 9 | 17. Eating less dinner and more breakfast is good. | Eating a good breakfast allows you to reduce the size of your lunch and your risk of diabetes. Eat less dinner because we rarely exercise at night. |
|  | 18. How do girls order takeout at work during weight loss? | When ordering takeout, pay attention to the type and proportion of food. |
| Week 10 | 19. Which exercise can reduce fat and increase muscle? | To reduce fat and increase muscle, you need to add anaerobic exercise to aerobic exercise (such as high-intensity interval training). |
|  | 20. Why don't you lose weight after exercising? | How long and how much you exercise, as well as what you eat, can affect your weight. |
| Week 11 | 21. Do these things, and have a beautiful sleep. | The standard of high quality sleep, the way to create a good sleep environment. |
|  | 22. Meditation before bed can improve sleep. | Introduce the concept of meditation, the benefits of meditation, and the steps of meditation. |
| Week 12 | 23. Learning to reduce stress and relax. | Reduce stress by exercising, speaking your mind, getting quality sleep, letting off steam, taking vitamin C supplements, and taking up hobbies. |
|  | 24. With the long-term treatment, they really cured PCOS. | Show some cases of PCOS patients who have successfully improved PCOS after adhering to lifestyle changes. |
