# Supplementary material for: Efficacy of WeChat-Based Digital Intervention Versus Metformin in Women With Polycystic Ovary Syndrome: Randomized Controlled Trial
Source: J Med Internet Res. 2024 Oct 2;26:e55883. doi: 10.2196/55883 (PMC11483258; doi:10.2196/55883)
Supplement: Multimedia Appendix 3 [file jmir_v26i1e55883_app3.docx]

**Multimedia Appendix 3: Satisfaction survey on digital intervention (n=35).**

| Questions | Yes, n (%) | No, n (%) | Uncertain, n (%) |
| --- | --- | --- | --- |
| 1. Is the overall experience of this WeChat mini-program satisfactory? | 28 (80.0%) | 4 (11.4%) | 3 (8.6%) |
| 2. Does this WeChat mini-program save your time? | 31 (88.6%) | 3 (8.6%) | 1 (2.9%) |
| 3. Has this WeChat mini-program solved your problem with PCOS? | 26 (74.3%) | 3 (8.6%) | 6 (17.1%) |
| 4. Compared with the outpatient clinic, does this WeChat mini-program explain PCOS-related knowledge more comprehensively? | 30 (85.7%) | 1 (2.9%) | 4 (11.4%) |
| 5. Would you recommend this WeChat mini-program to others? | 28 (80.0%) | 4 (11.4%) | 3 (8.6%) |
