# Supplementary material for: Efficacy of WeChat-Based Digital Intervention Versus Metformin in Women With Polycystic Ovary Syndrome: Randomized Controlled Trial
Source: J Med Internet Res. 2024 Oct 2;26:e55883. doi: 10.2196/55883 (PMC11483258; doi:10.2196/55883)

Multimedia Appendix 4: The adherence rate of the digital intervention group and metformin group.


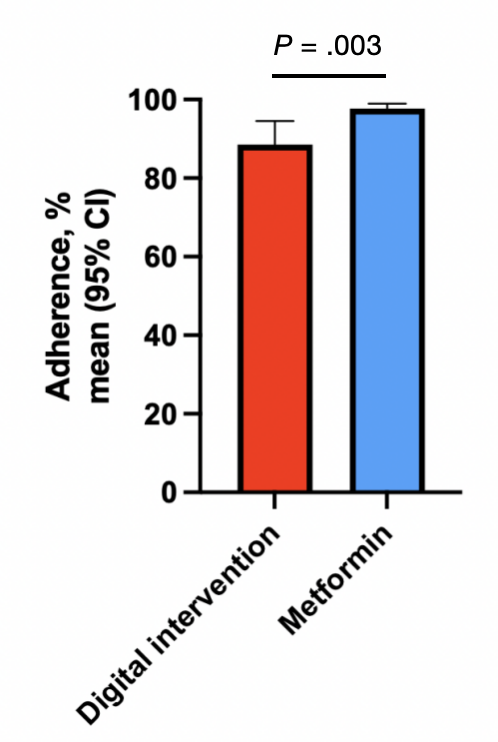

Supplement: Multimedia Appendix 4 [file jmir_v26i1e55883_app4.docx]
